# Correction: Large-scale releases and establishment of *w*Mel *Wolbachia* in *Aedes aegypti* mosquitoes throughout the Cities of Bello, Medellín and Itagüí, Colombia

**DOI:** 10.1371/journal.pntd.0012936

**Published:** 2025-03-11

**Authors:** Iván Darío Velez, Alexander Uribe, Jovany Barajas, Sandra Uribe, Sandra Ángel, Juan David Suaza-Vasco, Juan Sebastian Duran Ahumada, Maria Camila Mejia Torres, María Patricia Arbeláez, Eduardo Santacruz-Sanmartin, Lorena Duque, Luis Martínez, Tania Posada, Ana Cristina Patiño, Sandra Milena Gonzalez, Ana Lucía Velez, Jennifer Ramírez, Marlene Salazar, Sandra Gómez, Jorge E. Osorio, Inaki Iturbe-Ormaetxe, Yi Dong, Frederico C. Muzzi, Edwige Rances, Petrina H. Johnson, Ruth Smithyman, Bruno Col, Benjamin R. Green, Tibor Frossard, Jack Brown-Kenyon, D. Albert Joubert, Nelson Grisales, Scott A. Ritchie, Jai A. Denton, Jeremie R. L. Gilles, Katherine L. Anders, Simon C. Kutcher, Peter A. Ryan, Scott L. O’Neill

## Notice of republication

This article was republished on February 24, 2025, to correct the author list and add Juan Sebastian Duran Ahumada as the seventh author. Please download this article again to view the correct version. The originally published, uncorrected article and the republished, corrected article are provided here for reference.

## Supporting information

S1 File Originally published, uncorrected article. (PDF)

S2 File Republished, corrected article. (PDF)
