# Supplementary material for: Large-scale releases and establishment of wMel Wolbachia in Aedes aegypti mosquitoes throughout the Cities of Bello, Medellín and Itagüí, Colombia
Source: PLoS Negl Trop Dis. 2023 Nov 30;17(11):e0011642. doi: 10.1371/journal.pntd.0011642 (PMC10688688; doi:10.1371/journal.pntd.0011642)
Supplement: S1 Table — Participation activities are those where members of the community directly interact with WMP staff and partners. Communication activities are semi-targeted advertising. (DOCX) [file pntd.0011642.s001.docx]

**S1 Table. Engagement Activities.** Participation activities are those where members of the community directly interact with WMP staff and partners. Communication activities are semi-targeted advertising.

| **Activity** | **Bello** | **Medellín** | **Itagüí** |
| --- | --- | --- | --- |
| **Participation Activities** | | | |
| Meetings / Social Events (Participants) | 496 (26 thousand) | 2127 (127 thousand) | 214 (23 thousand) |
| Leaders Trained | 106 | 240 | – |
| Plays | 5 | 17 | – |
| Teachers Trained | 30 | 225 | – |
| **Communications Activities** | | | |
| Media Appearances | 350 | | 26 |
| Facebook Posts (Audience) | 754 (1.1 million) | | 74 (23 thousand) |
| Twitter Posts (Audience) | 571 (286 thousand) | | 32 |
| Instagram Posts (Audience) | 165 (35 thousand) | | 52 |
| Website Publications (Visits) | 191 (84 thousand) | | Global Website |
