# Supplementary material for: Large-scale releases and establishment of wMel Wolbachia in Aedes aegypti mosquitoes throughout the Cities of Bello, Medellín and Itagüí, Colombia
Source: PLoS Negl Trop Dis. 2023 Nov 30;17(11):e0011642. doi: 10.1371/journal.pntd.0011642 (PMC10688688; doi:10.1371/journal.pntd.0011642)
Supplement: S2 Table — (DOCX) [file pntd.0011642.s002.docx]

**S2 Table. *Wolbachia*-infected *Aedes aegypti* Mosquito Release Numbers.**

| **Comuna** | **Total Area (km^2^)** | **Release Area (km^2^)** | **Residents** | **Release Method** | **Phase 1** | | | | **Phase 2** | | | |
| --- | --- | --- | --- | --- | --- | --- | --- | --- | --- | --- | --- | --- |
|  |  |  |  |  | **Release weeks** | **Release total** | **Release average per week** | **Total mosquitoes per km^2^** | **Release weeks** | **Release Total** | **Release average per week** | **Total mosquitoes per km^2^** |
| **Bello** | | | | | | | | | | | | |
| Altos de Niquía | 1.35 | 1.1 | 85,828 | Adult | 14 | 640,075 | 45,720 | 581,886 | 15 | 594,087 | 39,606 | 540,080 |
| Bellavista | 1.88 | 1.3 | 56,949 | Adult | 10 | 385,544 | 38,554 | 296,572 | 15 | 459,594 | 30,640 | 353,536 |
| Fontidueño | 1.52 | 0.76 | 21,838 | Adult | 12 | 285,144 | 23,762 | 375,189 | 27 | 731,281 | 27,084 | 962,215 |
| Guasimalito | 3.02 | 0.62 | 8,915 | Adult | 15 | 114,879 | 7,659 | 185,289 | 15 | 137,602 | 9,173 | 221,938 |
| La Cumbre | 2.03 | 1.33 | 54,026 | Adult | 10 | 374,654 | 37,465 | 281,695 | 21 | 708,002 | 33,714 | 532,333 |
| La Madera | 1.93 | 1.71 | 61,636 | Adult | 15 | 840,233 | 56,016 | 491,364 | 8 | 338,255 | 42,282 | 197,809 |
| Niquía | 2.44 | 1.71 | 46,251 | Adult | 15 | 524,026 | 34,935 | 306,448 | 15 | 592,909 | 39,527 | 346,731 |
| París | 1.3 | 1.26 | 60,142 | Adult; WMP Egg | 31 | 486,667 | 15,699 | 386,243 | – | – | – | – |
| Santa Ana | 2.18 | 1.43 | 20,248 | Adult | 14 | 257,919 | 18,423 | 180,363 | 16 | 309,660 | 19,354 | 216,544 |
| Suárez | 1.91 | 1.73 | 107,314 | Adult | 14 | 74,1251 | 52,947 | 428,469 | 16 | 931,743 | 58,234 | 538,581 |
| Zamora | 1.81 | 1.07 | 30,844 | Adult | 15 | 429,753 | 28,650 | 401,638 | 33 | 728,394 | 22,073 | 680,743 |
| **Medellín** | | | | | | | | | | | | |
| Belén | 9.00 | 7.14 | 227,794 | Adult | 1 | 156,390 | 156390 | 21,903 | 21 | 4,559,837 | 217,135 | 638,633 |
| Buenos Aires | 6.06 | 4.27 | 158,373 | Adult | – | – | – | – | 20 | 4,462,927 | 223,146 | 1,045,182 |
| Castilla | 6.07 | 4.87 | 173,740 | Adult | – | – | – | – | 21 | 4,292,764 | 204,417 | 881,471 |
| Doce de Octubre | 3.85 | 3.64 | 224,461 | Adult | – | – | – | – | 22 | 3,701,166 | 168,235 | 1,016,805 |
| El Poblado | 14.40 | 8.89 | 150,465 | Adult | 15 | 4,495,313 | 299,687 | 505,660 | 11 | 3,454,287 | 314,026 | 388,560 |
| Guayabal | 7.29 | 5.07 | 109,735 | Adult | 15 | 2,462,831 | 164,189 | 485,765 | 31 | 4,775,022 | 154,033 | 941,819 |
| La América | 3.97 | 3.49 | 111,645 | Adult | – | – | – | – | 20 | 3,563,384 | 178,169 | 1,021,026 |
| La Candelaria | 7.36 | 6.49 | 98,904 | Adult | – | – | – | – | 23 | 4,845,486 | 210,673 | 746,611 |
| Laureles-  Estadio | 7.40 | 6.18 | 141,563 | Adult | 5 | 1,216,778 | 243,357 | 196,890 | 11 | 2,770,322 | 251,847 | 448,271 |
| Robledo | 9.47 | 6.11 | 200,004 | Adult | – | – | – | – | 20 | 4,849,729 | 242,486 | 793,736 |
| San Javier | 4.86 | 3.98 | 160,194 | Adult | – | – | – | – | 21 | 1,861,703 | 88,653 | 467,764 |
| Villa Hermosa | 5.72 | 4.31 | 159,524 | Adult | 6 | 714,561 | 119,094 | 165,791 | 26 | 3,636,013 | 139,847 | 843,623 |
| **Case Control** | | | | | | | | | | | | |
| Aranjuez A | 2.32 | 2.12 | 105,873 | Adult; Natural | 15 | 1,213,904 | 80,927 | 572,596 | 31 | 1,907,104 | 61519 | 899,578 |
| Aranjuez B | 2.56 | 2.04 | 82,021 | Adult; Natural | – | – | – | – | 13 | 1,421,984 | 109383 | 697,053 |
| Manrique A | 2.42 | 1.89 | 87,984 | Adult; Natural | 15 | 1,076,602 | 71,773 | 569,631 | 25 | 1,349,500 | 53980 | 714,020 |
| Manrique B | 2.68 | 2.35 | 97,347 | Adult; Natural | – | – | – | – | 13 | 1,928,501 | 148346 | 820,640 |
| Popular | 3.10 | 2.8 | 151,283 | Adult; Natural | – | – | – | – | 13 | 2,051,609 | 157816 | 732,719 |
| Santa Cruz | 2.19 | 2.08 | 129,417 | Adult; Natural | 15 | 1,224,891 | 81,659 | 588,892 | 25 | 1,907,952 | 76,318 | 917,285 |
| **Itagüí** | | | | | | | | | | | | |
| Itagüí | 12.73 | 11.36 | 274,987 | Adult; Community Egg; WMP Egg | – | – | – | – | 43 | 7,577,959 | 178,169 | 667,073 |

Non-residential areas such as parks, airports and industrial zones were excluded from *Wolbachia*-infected mosquito deployments. Releases are divided by phase: P1 are the initial releases; P2 are releases after insecticide resistance matching. Release weeks denote the total number of weeks that release occurred in. comuna highlighting denotes release strategy. Release Method is the strategy for the release of *w*Mel-infected *Ae. aegypti* and are described in the Materials and Methods. These methods are: ‘Adult’ releases involving mosquito releases from vehicles or on-foot; ‘WMP Egg’ releases that involved the establishment of mosquito release containers (MRCs) by WMP employees; ‘Community Egg’ releases that involved giving community members MRCs and capsules containing mosquito eggs to establish in their homes; ‘Natural’ releases that involved depositing mosquito eggs into natural breeding site
