# Supplementary figures and images for: Large-scale releases and establishment of wMel Wolbachia in Aedes aegypti mosquitoes throughout the Cities of Bello, Medellín and Itagüí, Colombia
Source: PLoS Negl Trop Dis. 2023 Nov 30;17(11):e0011642. doi: 10.1371/journal.pntd.0011642 (PMC10688688; doi:10.1371/journal.pntd.0011642)

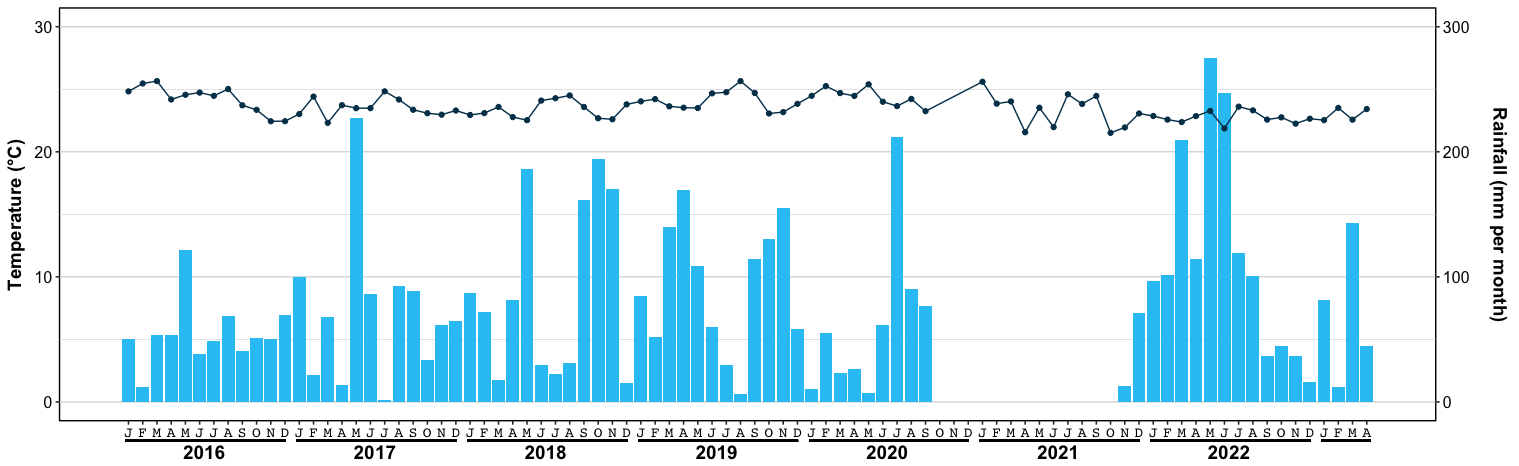

Supplement: S1 Fig — The average daily temperature per month is indicated in dark blue. The cumulative monthly rainfall (mm) is indicated in light blue. Data is derived from the weather station located at the Medellín Olaya Herrera Airport and extracted from the National Climate Data Centre (USA). Precipitation data from 2020 to 2021 was absent from the available data set. (TIF) [file pntd.0011642.s003.tif]

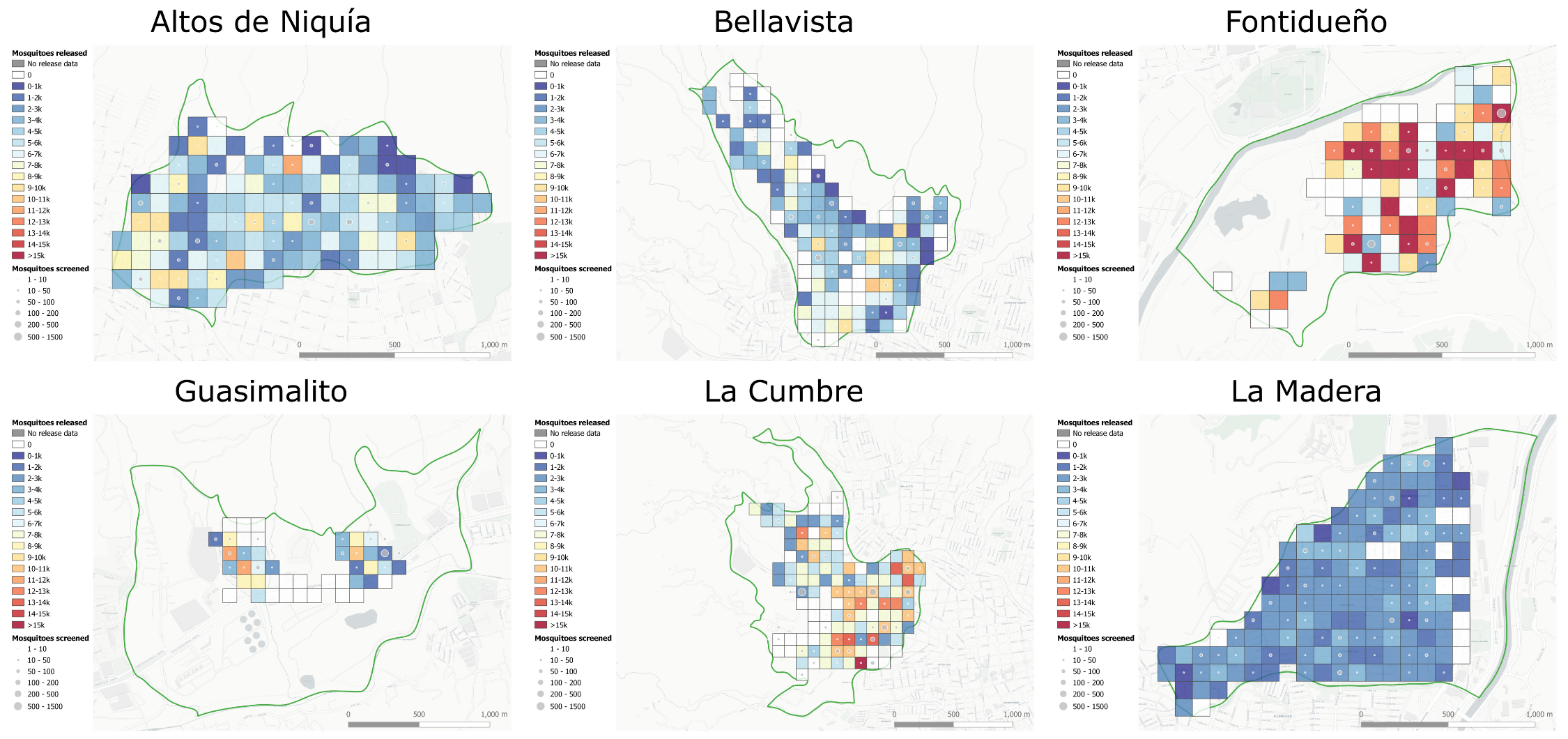

Supplement: S2 Fig — Each comuna was divided into a 100m2 grid with grid squares lacking mosquito releases omitted (maps produced in QGIS version 3.16.1 using administrative boundaries for the municipal government of Bello (https://www.datos.gov.co/Ordenamiento-Territorial/Divisi-n-Pol-tico-Administrativa-Barrios-Bello-Ant/pnhh-ccwd) and OpenMapTiles basemap layer (https://openmaptiles.org/) with CARTO light design (https://carto.com/)). Release gradient was determined by using GPS coordinates of each release event and assigning the number of wMel-infected mosquitoes to a corresponding grid square. Monitoring numbers were determined in the same way. (TIF) [file pntd.0011642.s004.tif]

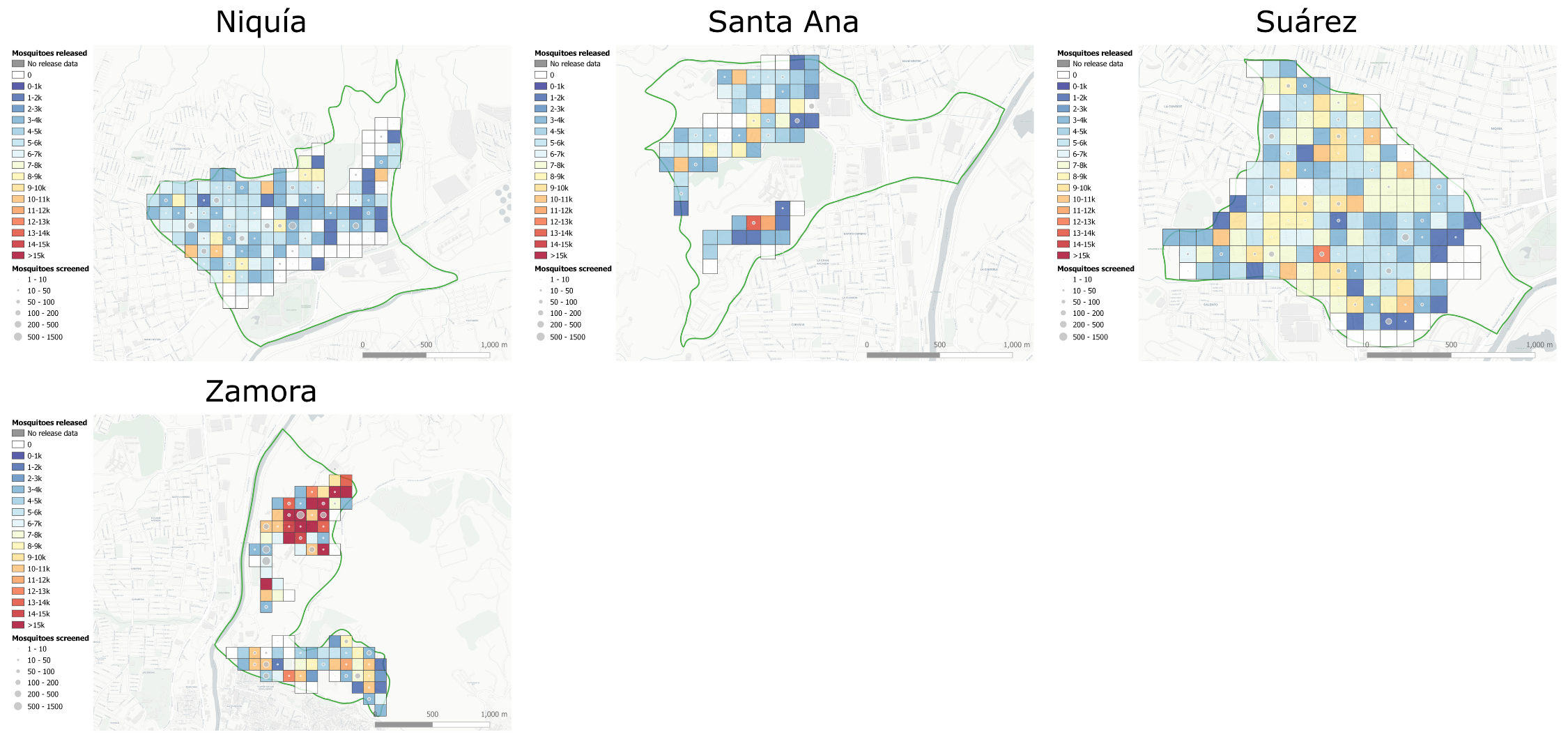

Supplement: S3 Fig — Each comuna was divided into a 100m2 grid with grid squares lacking mosquito releases omitted (maps produced in QGIS version 3.16.1 using administrative boundaries for the municipal government of Bello (https://www.datos.gov.co/Ordenamiento-Territorial/Divisi-n-Pol-tico-Administrativa-Barrios-Bello-Ant/pnhh-ccwd) and OpenMapTiles basemap layer (https://openmaptiles.org/) with CARTO light design (https://carto.com/)). Release gradient was determined by using GPS coordinates of each release event and assigning the number of wMel-infected mosquitoes to a corresponding grid square. Monitoring numbers were determined in the same way. (TIF) [file pntd.0011642.s005.tif]

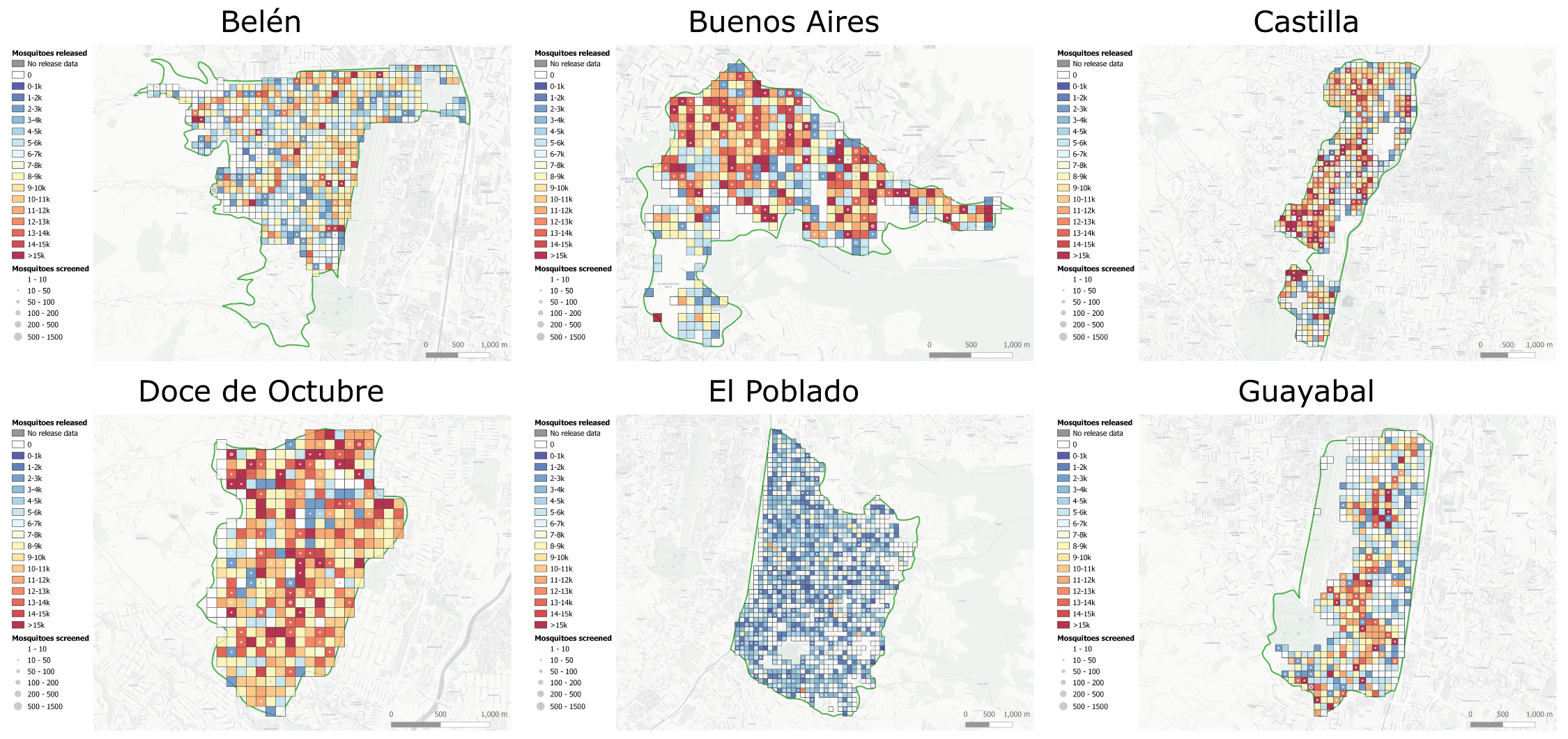

Supplement: S4 Fig — Each comuna was divided into a 100m2 grid with grid squares lacking mosquito releases omitted (maps produced in QGIS version 3.16.1 using administrative boundaries for the municipal government of Medellín (https://data.metabolismofcities.org/library/maps/35283/view/) and OpenMapTiles basemap layer (https://openmaptiles.org/) with CARTO light design (https://carto.com/)). Release gradient was determined by using GPS coordinates of each release event and assigning the number of wMel-infected mosquitoes to a corresponding grid square. Monitoring numbers were determined in the same way. (TIF) [file pntd.0011642.s006.tif]

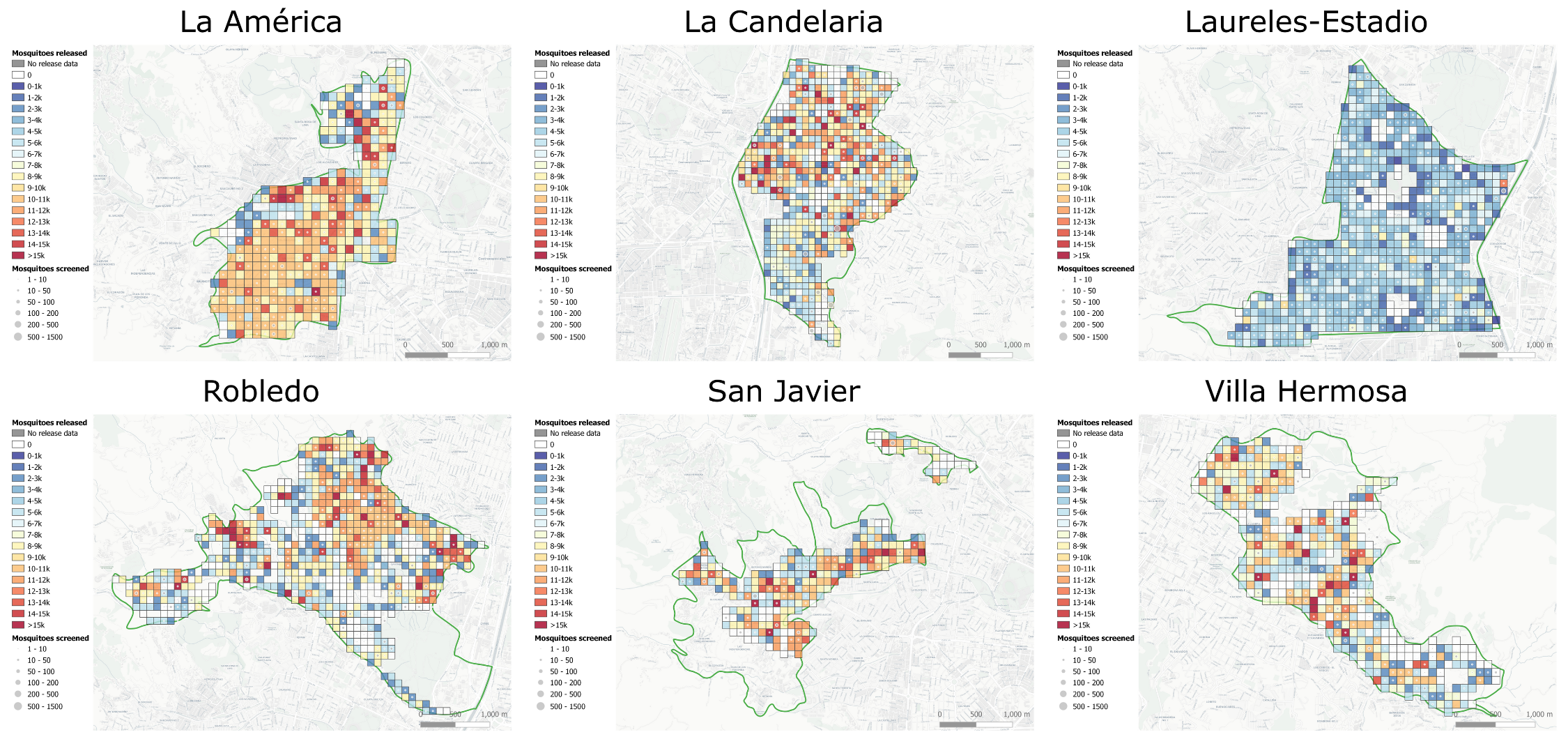

Supplement: S5 Fig — Each comuna was divided into a 100m2 grid with grid squares lacking mosquito releases omitted (maps produced in QGIS version 3.16.1 using administrative boundaries for the municipal government of Medellín (https://data.metabolismofcities.org/library/maps/35283/view/) and OpenMapTiles basemap layer (https://openmaptiles.org/) with CARTO light design (https://carto.com/)). Release gradient was determined by using GPS coordinates of each release event and assigning the number of wMel-infected mosquitoes to a corresponding grid square. Monitoring numbers were determined in the same way. (TIF) [file pntd.0011642.s007.tif]

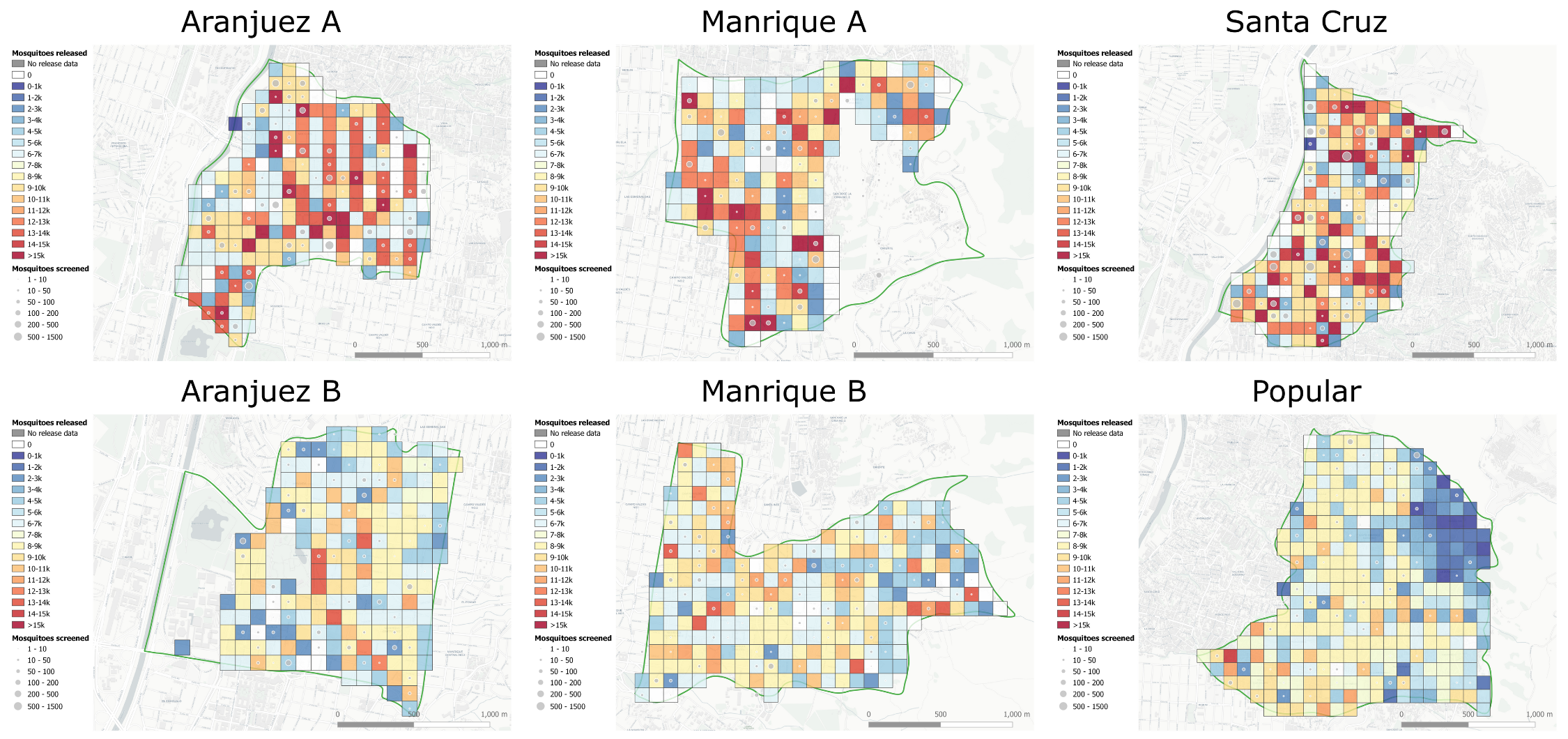

Supplement: S6 Fig — Each comuna was divided into a 100m2 grid with grid squares lacking mosquito releases omitted (maps produced in QGIS version 3.16.1 using administrative boundaries for the municipal government of Medellín (https://data.metabolismofcities.org/library/maps/35283/view/) and OpenMapTiles basemap layer (https://openmaptiles.org/) with CARTO light design (https://carto.com/)). Release gradient was determined by using GPS coordinates of each release event and assigning the number of wMel-infected mosquitoes to a corresponding grid square. Monitoring numbers were determined in the same way. (TIF) [file pntd.0011642.s008.tif]

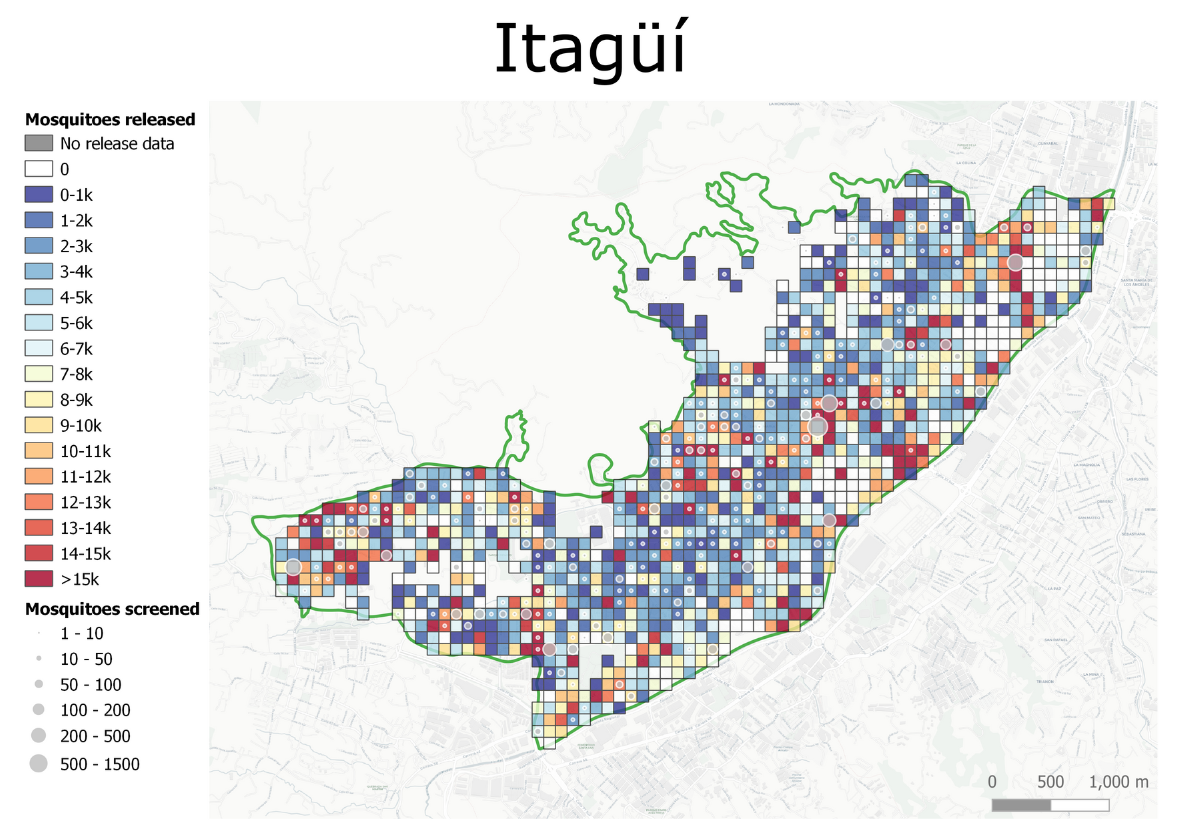

Supplement: S7 Fig — The area was divided into a 100m2 grid with grid squares lacking mosquito releases omitted (map produced in QGIS version 3.16.1 using administrative for the municipal government of Itagüí (https://www.datos.gov.co/Ordenamiento-Territorial/Localizaci-n-Geogr-fica-de-los-Barrios-del-Municip/didi-drqa)) and OpenMapTiles basemap layer (https://openmaptiles.org/) with CARTO light design (https://carto.com/)). Release gradient was determined by using GPS coordinates of each release event and assigning the number of wMel-infected mosquitoes to a corresponding grid square. Monitoring numbers were determined in the same way. (TIF) [file pntd.0011642.s009.tif]

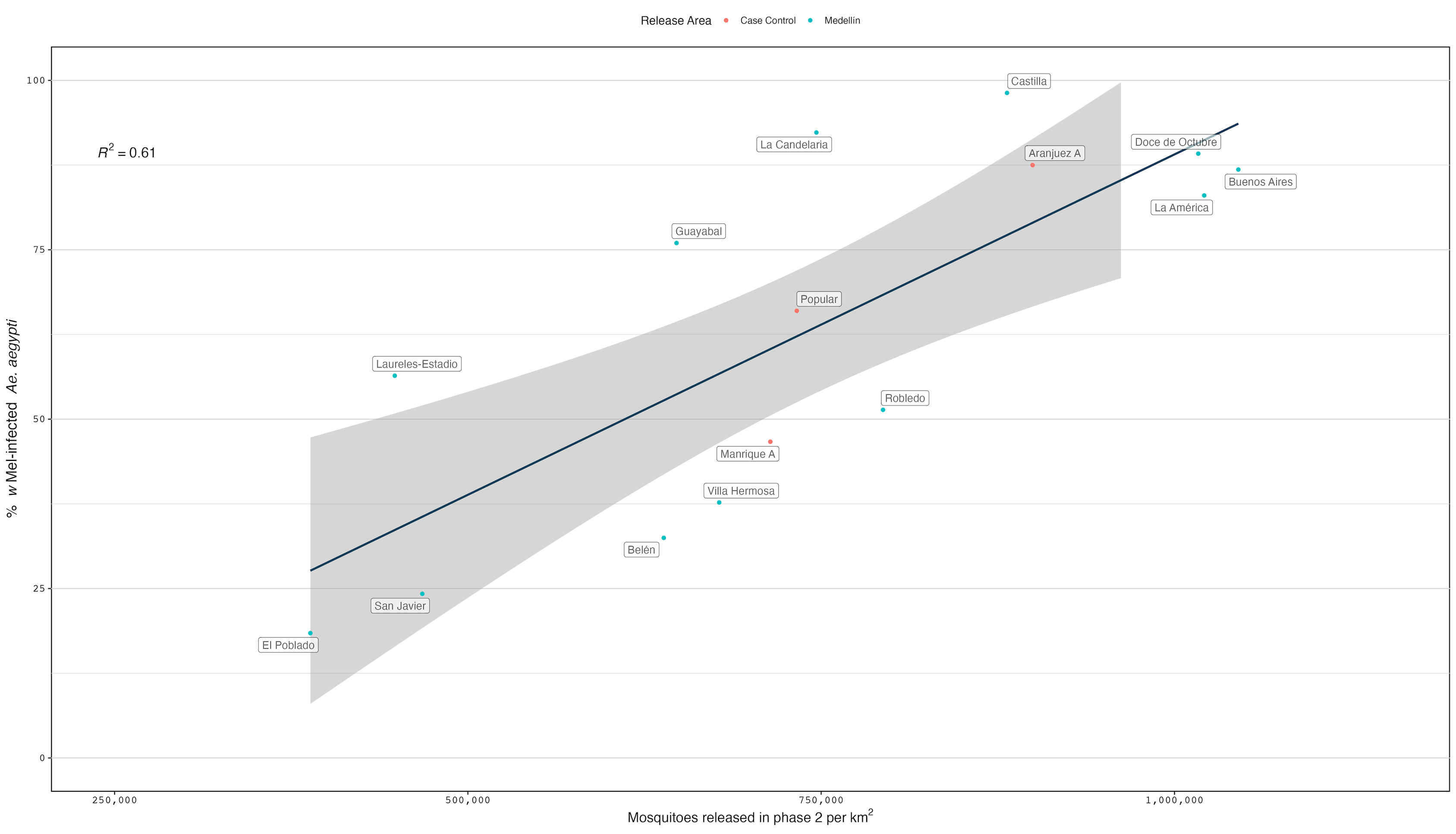

Supplement: S8 Fig — The predicted line from a linear model fit between prevalence of wMel at time of last monitoring and the number of wMel-infected mosquitoes released per km2 in a given area. Values are provided in S2 Table. (TIF) [file pntd.0011642.s010.tif]
