# Supplementary material for: Correction: Large-scale releases and establishment of wMel Wolbachia in Aedes aegypti mosquitoes throughout the Cities of Bello, Medellín and Itagüí, Colombia
Source: PLoS Negl Trop Dis. 2025 Mar 11;19(3):e0012936. doi: 10.1371/journal.pntd.0012936 (PMC11896058; doi:10.1371/journal.pntd.0012936)
Supplement: S1 File — (PDF) [file pntd.0012936.s001.pdf]

RESEARCH ARTICLE

# Large-scale releases and establishment of *w*Mel *Wolbachia* in *Aedes aegypti* mosquitoes throughout the Cities of Bello, Medellín and Itagüí, Colombia

**Iván Darío Velez[1], Alexander Uribe[1], Jovany Barajas[1], Sandra Uribe[1], Sandra Ángel[1], Juan David Suaza-Vasco[1], Maria Camila Mejia Torres[1], María Patricia Arbeláez[1], Eduardo Santacruz-Sanmartin[1], Lorena Duque[1], Luis Martínez[1], Tania Posada[1], Ana Cristina Patiño[1], Sandra Milena Gonzalez[1], Ana Lucía Velez[1], Jennifer Ramírez[1], Marlene Salazar[1], Sandra Gómez[1], Jorge E. Osorio[2], Inaki Iturbe-Ormaetxe[2], Yi Dong[2], Frederico C. Muzzi[2], Edwige Rances[2], Petrina H. Johnson[2], Ruth Smithyman[2], Bruno Col[2], Benjamin R. Green[2], Tibor Frossard[2], Jack Brown-Kenyon[2], D. Albert Joubert[2], Nelson Grisales[2], Scott A. Ritchie[2], Jai A. Denton[2], Jeremie R. L. Gilles[2], Katherine L. Anders[2], Simon C. Kutcher[2], Peter A. Ryan[2]*, Scott L. O'Neill[2]**

**1** World Mosquito Program, Universidad de Antioquia, Medellín, Colombia, **2** World Mosquito Program, Monash University, Clayton, Australia

* peter.ryan@worldmosquito.org

OPEN ACCESS

**Citation:** Velez ID, Uribe A, Barajas J, Uribe S, Ángel S, Suaza-Vasco JD, et al. (2023) Large-scale releases and establishment of *w*Mel *Wolbachia* in *Aedes aegypti* mosquitoes throughout the Cities of Bello, Medellín and Itagüí, Colombia. PLoS Negl Trop Dis 17(11): e0011642. https://doi.org/10.1371/journal.pntd.0011642

**Editor:** Amy C. Morrison, University of California Davis School of Veterinary Medicine, UNITED STATES

**Received:** May 3, 2023

**Accepted:** September 5, 2023

**Published:** November 30, 2023

**Copyright:** © 2023 Velez et al. This is an open access article distributed under the terms of the Creative Commons Attribution License, which permits unrestricted use, distribution, and reproduction in any medium, provided the original author and source are credited.

**Data Availability Statement:** The supporting data is available at 'https://doi.org/10.6084/m9.figshare.24045993.v1

**Funding:** This work was supported by a grant from the Bill & Melinda Gates Foundation (OPP1159497 to SLO), the Wellcome Trust working in partnership with the UK Department for International Development (Grant 102591/Z/13/A to SLO) and the US Agency for International

## Abstract

### Background

The *w*Mel strain of *Wolbachia* has been successfully introduced into *Aedes aegypti* mosquitoes and has been shown to reduce the transmission of dengue and other *Aedes*-borne viruses. Here we report the entomological results from phased, large-scale releases of *Wolbachia* infected *Ae*. *aegypti* mosquitoes throughout three contiguous cities located in the Aburrá Valley, Colombia.

### Methodology/principal findings

Local *w*Mel *Wolbachia*-infected *Ae*. *aegypti* mosquitoes were generated and then released in an initial release pilot area in 2015–2016, which resulted in the establishment of *Wolbachia* in the local mosquito populations. Subsequent large-scale releases, mainly involving vehicle-based releases of adult mosquitoes along publicly accessible roads and streets, were undertaken across 29 comunas throughout Bello, Medellín and Itagüí Colombia between 2017–2022. In 9 comunas these were supplemented by egg releases that were undertaken by staff or community members. By the most recent monitoring, *Wolbachia* was found to be stable and established at consistent levels in local mosquito populations (>60% prevalence) in the majority (67%) of areas.

Development (AID-OAA-A-16-00081 to SLO). The funders had no role in study design, data collection and analysis, decision to publish, or preparation of the manuscript.

**Competing interests:** The authors have declared that no competing interests exist.

## Conclusion

These results, from the largest contiguous releases of *w*Mel *Wolbachia* mosquitoes to date, highlight the operational feasibility of implementing the method in large urban settings. Based on results from previous studies, we expect that *Wolbachia* establishment will be sustained long term. Ongoing monitoring will confirm *Wolbachia* persistence in local mosquito populations and track its establishment in the remaining areas.

## Author summary

The introduction of the naturally occurring *w*Mel *Wolbachia* strain into *Aedes aegypti* mosquitoes has been shown to reduce the ability of the mosquitoes to transmit dengue and other viruses. Following engagement with communities to gain acceptance and support, a series of large-scale releases of *Ae. aegypti* mosquitoes that contained *w*Mel *Wolbachia*, were undertaken across the cities of Bello, Medellín and Itagüí in Colombia. These releases were undertaken under operational conditions with the aim of rapidly scaling the intervention in response to the Zika virus crisis. Mosquito populations were monitored during and after releases to determine the levels of *Wolbachia* and whether it persisted in the local mosquitoes. *Wolbachia* was found to be stable and established at consistent levels in local mosquito populations in the majority of areas. On-going monitoring in these areas will determine whether *Wolbachia* persists and also whether it establishes at a high level in the remaining areas. This intervention forms the basis of an epidemiological study to assess the impact of operational deployment of *w*Mel *Wolbachia* on the reduction of the incidence of notified dengue cases and virologically-confirmed dengue.

## Introduction

*Aedes aegypti* mosquitoes containing the *w*Mel *Wolbachia* strain have been shown in laboratory studies to have a reduced ability to transmit a range of viruses including dengue, Zika, chikungunya, yellow fever and Mayaro viruses [1–4]. Field trials involving releases of *w*Mel *Wolbachia* infected *Ae. aegypti* mosquitoes have shown that *Wolbachia* can be deployed and established in local mosquito populations [5–15]. In areas where *w*Mel *Wolbachia* has been established at high levels in local mosquito populations, dengue incidence has been significantly reduced, resulting in near elimination of local dengue transmission in northern Australia [11,12]; 73% reduction in dengue incidence in a quasi-experimental trial in Yogyakarta, Indonesia [10]; 77.1% reduction in dengue incidence in a cluster randomised trial in Yogyakarta, Indonesia [14]; and 69% reduction in dengue incidence, 56% reduction in chikungunya incidence, and 37% reduction in Zika incidence, in Niterói, Brazil [16].

In large-scale *Wolbachia* releases covering 86.8 $km^2$ and 890,000 people in Rio de Janeiro, Brazil, the establishment of *Wolbachia* within the first two years post-release was heterogeneous and with the prevalence of *Wolbachia* ranging from $\sim$30% to >80% among neighbourhoods [17]. The heterogeneity in *Wolbachia* establishment was thought to be due to the complex urban settings, including significant spatial variation in the baseline *Ae. aegypti* populations and limited access to some areas, such as favela communities. However, the initial *Wolbachia*-infected Brazilian release strain was insecticide sensitive [5]. This was found to inhibit the spread of *Wolbachia* and required the creation of a new strain with an insecticide

resistance profile closely matching local mosquitoes [5]. Despite intermediate *Wolbachia* infection prevalence in mosquitoes, *Wolbachia* releases still resulted in significant reductions in the incidence of both dengue and chikungunya in Rio de Janeiro [17] and in the neighbouring city of Niterói [16].

To further develop and evaluate the scalability of *w*Mel *Wolbachia* releases as an effective intervention for use in large urban settings, a series of *Wolbachia* mosquito releases were undertaken across the cities of Bello, Medellín and Itagüí in the Aburrá Valley, Colombia. These releases commenced with an initial small-scale pilot release in 2015 in the neighbourhood of Paris in Bello. Following the declaration of Zika as a public health emergency by the World Health Organization (WHO) [18] WHO assessed the available evidence for *Wolbachia* and determined that it warranted the pilot deployment of *Wolbachia* under operational conditions, including monitoring and evaluation and generation of evidence on its effectiveness [19]. Following this recommendation, *Wolbachia* releases were expanded initially across Bello (2016–2017), followed then by Medellín (2018–2021) and Itagüí (2019–2020). These large-scale releases were undertaken under operational conditions, mainly involving the releases of *Wolbachia* infected adult *Ae. aegypti* from vehicles, with the goal of covering areas as quickly as possible using a standardised release method. Supplementary releases of *Wolbachia* infected *Ae. aegypti* eggs using mosquito release containers were undertaken in some areas. Entomological outcomes were monitored by collection of mosquitoes using BG Traps and indoor Prokopack aspirator collections [20] and testing of these mosquitoes for *Wolbachia* infection [11,21]. Here we describe the entomological outcomes of these releases.

## Methods

### Ethics statement

a. Written approvals were obtained from the Bioethics Committee of the Research Headquarters of the University of Antioquia and the Ethics and Research Committee of the University IPS.

b. Bioethics Committee of the Research Headquarters of the University of Antioquia (Releases in Paris neighborhood, Approval number 13-05-514); Bioethics Committee of the Research Headquarters of the University of Antioquia (Releases in Bello, Approval number 05-15-2014); Ethics and Research Committee of the University IPS (Releases in Bello and Medellín, Approved 14 January 2017, ratified 25 October 2017).

c. No human participants or donors were involved in any activities.

### Intervention area

*Wolbachia* mosquito releases were undertaken across three municipalities (Bello, Medellín, Itagüí) located in the Aburrá Valley, within the Department of Antioquia in the northeast of Colombia. The three municipalities contain a combined population of 3.3 million people [22] and cover an area of 135 km$^2$. The metropolitan areas, located mainly within Medellín and Bello were located between 1400–1500 m above sea level, with the remaining areas located on the Eastern and Western sides of the valley at elevations between 1500–2100 m. Due to the high elevation and proximity to the equator, the municipalities experience a highly stable, warm year-round climate with average monthly daily temperatures ranging between 21.8 to 23.1˚C (S1 Fig) [23]. Each city is divided into administrative units called comunas, which were used for the purposes of *w*Mel mosquito release planning and reporting of entomological and

public health outcomes in Bello (11 comunas) and Medellín (16 comunas, two of which were divided in half giving 18 areas) (Fig 1). Itagüí has six comunas, but was treated as a single unit for operational and monitoring purposes.

## Community engagement

**Paris neighbourhood pilot releases.** For the *Wolbachia* releases in the Paris neighbourhood, community engagement activities followed those previously described [8]. This included consultation with key stakeholders and community groups, one-on-one meetings, displays at community events and centres and door-knocking. Prior to commencement of releases, residents were surveyed. The outcomes, timing and acceptance rates of all surveys are provided in Table 1.

**Large-scale public acceptance across Bello, Medellín and Itagüí.** For *Wolbachia* mosquito releases that were undertaken between 2017–2021, community engagement activities followed the Public Acceptance Model (PAM) as previously described [11,24,25]. To cover three million residents, the PAM method was modified to facilitate this expanded scope. The process included:

1. Raising broad community and stakeholder awareness across the release areas. Communication and community outreach campaigns were undertaken prior to commencement of releases and included advertising on billboards, placards, television, radio, social media, distribution of pamphlets to households, and attendance at community events. Engagement activities were targeted, temporally and spatially, to ensure project socialisation prior to support surveys. However, the nature of these activities would mean residents outside of target areas would also be exposed to the campaign. A summary of these activities is provided in S1 Table. These activities continued throughout the release period and served to provide updates to the community.
2. Quantitative surveys to assess community support. Cross-sectional surveys were undertaken by independent consultants to understand the levels of knowledge (dengue, *Wolbachia*), acceptance for releases and preferred methods for dissemination of information. The outcomes, timing and acceptance rates of all surveys are provided in Table 1.
3. Establishment of an issues management system. To allow community members to contact the project with any questions and concerns, a complaints, claims and requests system was established that facilitated and recorded community feedback. This allowed continuous monitoring of community sentiment and open lines of communication while also allowing a rapid response to any concerns.
4. Community reference groups. In previous PAM implementations, single community reference groups (CRGs) typically covered the entire release city. However, with greater numbers of residents, a more granular approach was undertaken across the Aburrá Valley. In each comuna a CRG was established with representatives from the communities, including businesses, churches, schools, government bodies, media, health centres and the wider community. The members independently reviewed communications and community engagement activities, disseminated information to the wider community, and brought any issues or concerns to the attention of the project.

**Approval for releases.** Deployments of *Wolbachi*a-infected *Ae*. *aegypti* in Bello, Medellín and Itagüí were regulated as a research project in partnership with the Universidad de Antioquia, Colombia. In addition to regulatory approvals below, ethical review was undertaken by

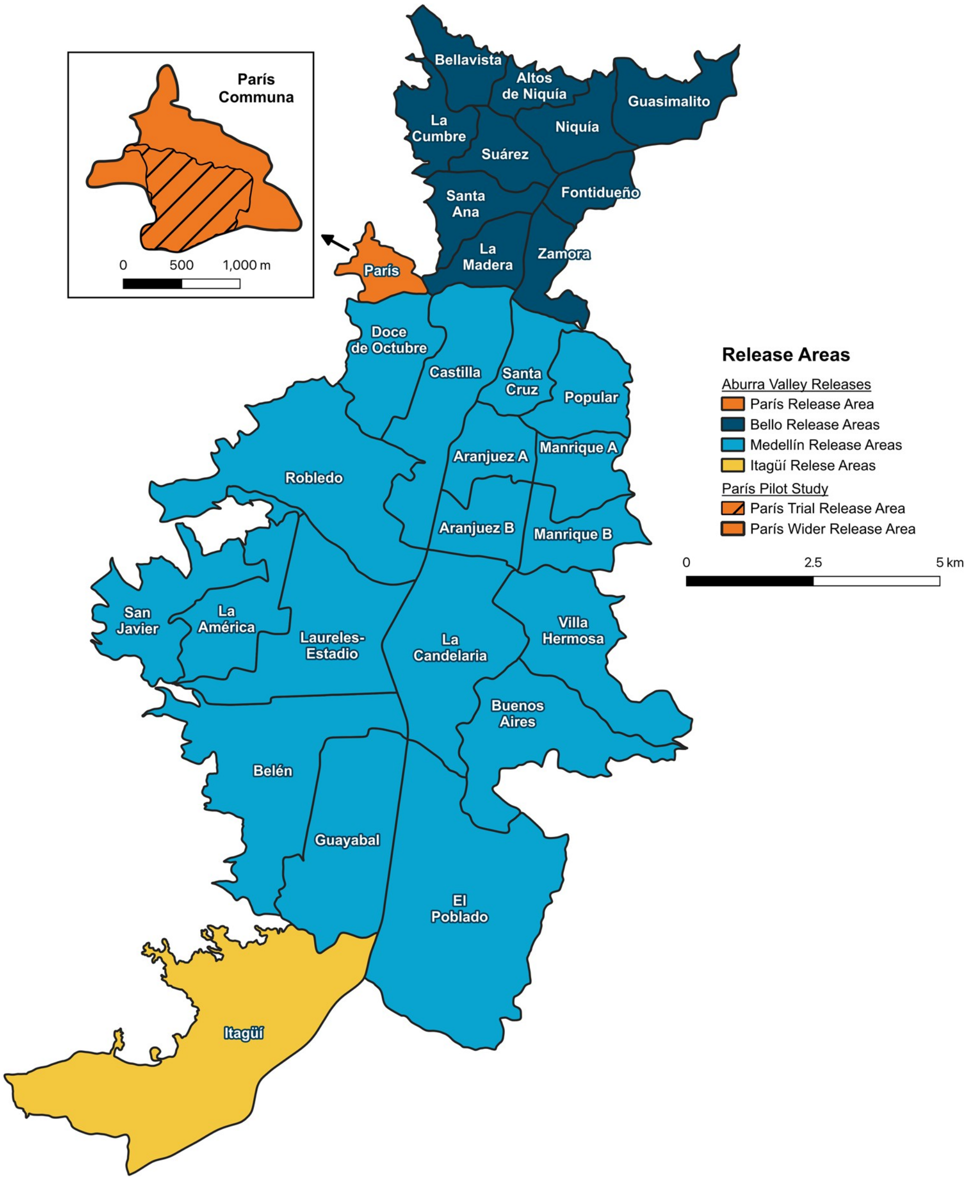


**Fig 1. Release areas within Bello, Medellín and Itagüí.** Dark blue, light blue and yellow shading denote Bello, Medellín and Itagüí respectively (map produced in QGIS version 3.28.3 using administrative boundaries for the municipal governments of Bello (https://www.datos.gov.co/Ordenamiento-Territorial/Divisi-n-Pol-tico-Administrativa-Barrios-Bello-Ant/pnhh-ccwd), Medellín (https://data.metabolismofcities.org/library/maps/35283/view/), and Itagüí (https://www.datos.gov.co/Ordenamiento-Territorial/Localizaci-n-Geogr-fica-de-los-Barrios-del-Municip/didi-drqa)). The initial París release area is coloured orange with the insert showing a close up of the area. The striped pattern within the insert indicates the initial trial.

https://doi.org/10.1371/journal.pntd.0011642.g001

the Bioethics Committee of the Research Headquarters of the University of Antioquia and the Ethics and Research Committee of the University IPS. Following the success of an initial trial in Paris, Bello, approval for larger scale releases was obtained from several levels of government. This included the Health Secretary of Bello municipality (local level), Health Secretary of Medellín municipality (local level), Health Secretary of Itagüí (local level), Ministry of Health in Bogota (national level), Health Secretary of Area Metropolitana (a conglomerate of 10 municipalities in the area) and Governor of Antioquia (department level). The National

**Table 1. Public acceptance of implementing the *Wolbachia* method within Bello, Medellín and Itagüí.**

| Location | Survey | Respondents | Timing | Acceptance (Other) |
|---|---|---|---|---|
| París | Pre-release | 4,741 | January, 2014 –August, 2015 | 93% |
| Bello | Pre-release | 336 | January–May, 2017 | 80% (4%) |
| Medellín | Pre-release | 336 | April–June, 2017 | 87% (4%) |
| Itagüí | Pre-release | 404 | September, 2019 | 97% |

Acceptance is the percentage of respondents approving of implementation. Pre-release surveys were undertaken prior to mosquito deployment. Values were determined by the sampling of random households within potential release areas. Participants were provided with the option to provide no answer or to say they didn't know. Percentage of unsure responses denoted in brackets.

https://doi.org/10.1371/journal.pntd.0011642.t001

Authority on Environmental Licences was consulted for the initial pilot in París but did not require additional consultation for wider releases.

## Mosquito production

***w*Mel *Aedes aegypti* release lines.** For the initial Paris neighbourhood releases in 2015–2016, a local *w*Mel *Ae. aegypti* line (*w*Mel-COL) was created by backcrossing infected virgin females from the Cairns, Australia *w*Mel-infected *Ae. aegypti* line [1] to F2 uninfected males from a colony (WT2) established from material collected from the Paris and Altos de Niquia neighbourhoods. The uninfected wild-type mosquitoes were collected as eggs from 41 positive ovitraps from households homogeneously distributed in Paris and Altos de Niquia comunas. Eleven generations of backcrossing were undertaken, followed by regular introduction of wild-type males (10%) added to the broodstock cages. The colony was maintained and amplified until 2018.

In May 2018 a second *w*Mel *Wolbachia Ae. aegypti* line (*w*Mel-COL2) was created with the aim of more closely matching the pyrethroid resistance profile of the release line with the wild-derived material in Medellín release areas. We started with three generations of outcrossing females from *w*Mel-COL with wild-derived F1 males from Medellín comunas. Then, the mosquitoes were exposed to Permethrin-impregnated papers (0.75% Al) for 1 hr in tubes [26]. Surviving individuals were collected. Mosquitoes were bloodfed and the progeny were reared for 2 generations. Individuals were again exposed to Permethrin for 1 hr and surviving individuals were collected. Progeny from these females were reared to adults and virgin females were mated during the next 4 generations with wild-derived F1 males from a colony (WT2) established from mosquitoes collected from Popular, Aranjuez, Doce de Octubre, San Javier and Belén comuna.

Finally, after the F5, 2 consecutive rounds of selection for knockdown resistance (*kdr*) mutations selection were undertaken. Individuals were screened (both Broodstocks & WT) for *kdr* mutations (F1534C, V1016I). These two alleles are highly prevalent throughout the Americas [27], including Colombia generally and the Aburrá Valley specifically [28,29]. Allele-specific PCR was undertaken as previously described [30–32]. The double mutants were used to establish the *w*Mel-COL2 line.

**Mosquito rearing.** The *w*Mel-*Ae. aegypti* lines (*w*Mel-COL and *w*Mel-COL2) were maintained as previously described [11]. Briefly, 600–800 larvae were reared in 40 x 25 x 8 cm plastic trays containing 1.5 L of reverse osmosis (RO) water and fed on Tetramin Tropical Flakes (Tetra Holding Inc., Germany—77101). Pupae were sex sorted using a sex sorter described in previous publications [34,35] and were then transferred into 30 x 30 x 30 cm cages (BugDorm, MageView Science Co. Ltd., Taiwan) at a density of 800 to 1000 pupae per cage (ratio 3:1 females:males).

Female mosquitoes (5–7 days old) were blood fed weekly until repletion (usually 10–15 mins). Mosquitoes were fed using blood-soaked gauze pads or via Hemotek feeders (Hemotek Ltd, UK). We only used human blood, obtained from blood banks, which would have been discarded by not attending quality assurance policies (e.g., blood bags with insufficient volume etc). All blood-bank supplied blood used for mosquito feeding had been tested negative for Hepatitis B, Hepatitis C, Chagas disease, syphilis, HIV, and HTLV (Labmedico, Medellín, Colombia). In addition, blood was screened again for DENV from 2015 until early 2018 and from 2018 until the end of releases for DENV, CHIK and ZIKA by qRT-PCT [36].

A proportion of the eggs produced by these colonies were used for the subsequent broodstock generation [11]. Remaining eggs were used for mass production, as follows. Eggs were hatched into 130 x 29 x 3 cm trays containing 5 L of RO water, at densities of 10,000–15,000 eggs per tray. Larvae were fed with a liquid diet (62.1% tuna meal, 37.9% beef liver made up in RO water). Pupae were sex sorted and transferred to large mesh cages (90 x 90 x 20 cm) at a density of approximately 15,000 pupae per cage (ratio 3:1 females:males). Emergent mosquitoes were maintained on sucrose solution (10%), and fed on human blood as described above for three gonotrophic cycles. Eggs were collected from containers lined with filter paper, placed on absorbent paper towel and stored in sealed plastic bags for three days at 27˚C, after which time they were removed from the plastic bags and air dried under insectary conditions (27˚C, 80% relative humidity).

**Rearing of adult mosquitoes for releases.** Eggs from the mass production colony were hatched and reared to late instar/pupal stages as described above. Late instars and pupae (250) were then placed into individual plastic cups (200 mL) containing 40–50 mL of tap water. When approximately 90% of immatures had pupated, a mesh cover was placed on each cup and adults were maintained for 3–4 days on 20% sucrose solution. Release cups were transferred to crates for transport to the release site. Release cups were maintained under ambient temperature conditions for an average of 6 hrs during transfer from the insectary to the release locations.

**Preparation of eggs for releases.** For the initial pilot releases in Paris in 2015–2016 and the releases in Manrique A, Aranjuez A and Santa Cruz comuna in 2018–2019, eggs were harvested from colony cages on oviposition strips of red cotton duck cloth that were placed in adult cages for three to five days after blood-feeding. Once collected, the oviposition strips were then dried and stored at 80% relative humidity until required [11]. Prior to releases, the density of eggs/cm on each strip was estimated to determine the length of egg strip to be cut to obtain approximately 100 eggs for subsequent use in the Mosquito Release Containers (MRCs) or for placement in natural, immature development sites.

In the Itagüí releases, eggs were gently brushed from the egg papers and were then passed through a 300–400 μm sieve to remove any body parts. Eggs were weighted using a scale accurate to +/- 1 mg, and the numbers of eggs were then estimated assuming an average egg weight of 8.8 μg per egg. The hatch rate of each batch of eggs was determined by aliquoting 5 replicates of 200 eggs each into 50 ml tubes each containing 40 ml of deoxygenated tap water and 32 mg of ground TetraMin Tropical Flakes (Tetra Holding Inc., Germany—77101). After 5 hours, the contents were transferred to trays containing 400 mL of tap water along with 40mg of TetraMin Tropical flakes. After 48 hrs the numbers of immatures were counted, and the average hatch rate percent was calculated. Egg capsule contents were prepared by mixing the following w/w: 50% tuna meal, 35% beef liver powder and 15% baker's yeast. Each capsule contained 260 mg of diet and 150 viable eggs as determined by the above egg hatch assessment. Larval diet was passed through a 425 μm sieve and then mixed with the eggs in a 12 L container by rotating the container for 2–3 minutes. Capsules (HPMC [hydroxypropylmethyl] cellulose size 00, FARMACAPSULAS, Barranquilla, Colombia) were then filled with the diet and egg

mixture using a manual capsule filler (Manual Capsule Filler Machine, Model no. CN-240CL, CapsulCN, Zhejiang, China) and then stored in sealed plastic containers at 18˚C.

**Diagnostic testing of samples for *Wolbachia*.** Colony and field collected mosquitoes were screened for *Wolbachia* using TaqMan qPCR on a Roche LightCycler 480 using an internally controlled qualitative assay for the presence or absence of *Wolbachia* as previously described [11,21]. Cycling conditions were: x1 95˚C for 5 minutes, x45 95˚C for 10 seconds, 60˚C for 15 seconds, 72˚C for 1 second with single acquisition and x1 40˚C for 10 seconds. *Wolbachia* was detected using WSP primers (F: 5'-CATTGGTGTTGGTGTTGGTG-3', R: 5'-ACACCAGCTTTTACTTGACCAG-3' with probe: 5'-LC640-TCCTTTGGAACCCGCTGT-GAATGA-IowaBlack-3'). *Ae. aegypti* RpS17 reference detected with primers F: 5'-TCCGTGGTATCTCCATCAAGCT-3', R: 5'-CACTTCCGGCACGTAGTTGTC-3' and probe 5'FAM- CAGGAGGAGGAACGTGAGCGCAG-BHQ1-3).

For quality assurance of the mosquito colonies, a total of 10 adult mosquitoes were randomly sampled from each cage at four to five days after blood-feeding, and were screened for DENV and CHIKV by qRT-PCT [36]. Primer and probe sequences are as follows; pan-DENV F: AAGGACTAGAGGTTAGAGGAGACCC and R: CGTTCTGTGCCTGGAATGATG, with probe 5'-Lc640 (or Cy5)- AACAGCATATTGACGCTGGGAGAGACCAGA- Iowablack -3' and CHIKV F:

5'-AAGCTYCGCGTCCTTTACCAAG3', R: 5'-CCAAATTGTCCYGGTCTTCCT-3' with probe 5'-HEX-CCAATGTCYTCNGCCTGGACACCTT- BHQ1-3'. RNA underwent one freeze-thaw cycle with qRT-PCR reaction performed using the Lightcycler Multiplex RNA Virus Master kit (Roche) with the following conditions; 50˚C for 10 mins, 95˚C for 30 sec, followed by 45 cycles of 95˚C for 3 sec, 60˚C for 30 sec, 72˚C for 1 sec and 1 cycle of 40˚C for 1 sec.

***Wolbachia* mosquito line fitness testing.** The *w*Mel-COL *Wolbachia* mosquito line was characterised in terms of key fitness traits including adult female fecundity, egg hatch rate, *Wolbachia* infection rate, cytoplasmic incompatibility, *Wolbachia* maternal transmission efficiency, insecticide susceptibility using previously described methods [1,21,26]. Fecundity was assessed with a total of 50 blood-fed female mosquitoes. Egg hatch rates were determined by immersing egg strips in trays containing 250mL of water and a small amount of larval diet, after which egg papers were dried down and stored for 3 days before immersing a second time. The numbers of larvae divided by the number of eggs from the first and second hatch were combined to determine the hatch rate of eggs. Cytoplasmic incompatibility (CI) was tested by reciprocally crossing the *w*Mel-COL line with the uninfected wild-type Paris neighbourhood (F0 or F1) line. Hatch rates in the compatible and incompatible crosses were compared. For the maternal transmission experiments, *Wolbachia* infected virgin females were mated with wild-type F2 males over a 24-hour period. After 24 h a human blood meal was provided, and individual engorged females were placed into oviposition cups. Eggs were collected from each cup and adult females and progeny were (n = 50) were processed for *Wolbachia* infection using the TaqMan qPCR as described above. The *w*Mel-COL release line was found to induce CI in uninfected mosquitoes, transmit *Wolbachia* from mother to offspring and have acceptable fecundity and egg hatch rates (Table 2).

The insecticide susceptibility of the *w*Mel-COL *Wolbachia* mosquito line was assessed using previously described methods [26]. Insecticide type and concentrations were in line with recommendations for *Ae. aegypti* mosquitoes and followed the WHO standard bioassay method [26]. Susceptible mosquitoes from the Rockefeller reference strain were used for positive and negative controls. Wild-type F0 or F1 *Ae. aegypti* mosquitoes collected from the Paris neighbourhood were also used as controls. In 2018, a second *w*Mel *Wolbachia Ae. aegypti* line was generated (*w*Mel-COL2), and this was screened for permethrin resistance.

**Table 2. *w*Mel-infected *Aedes aegypti* lines for release in Bello, Medellín and Itagüí.**

| Release line | Characteristic | Description | | | |
|---|---|---|---|---|---|
| *w*Mel-COL | Backcrossing source | París and Altos de Niquia comunas | | | |
| | Backcrossing method | Eleven generations of backcrossing and followed by introduction of 10% wild-type males added to cages each generation. | | | |
| | *Wolbachia* infection rate | Percentage of offspring with *w*Mel; *w*Mel-infected female x uninfected male; 100% | | | |
| | Fecundity | Eggs per Iso-female; 50 females per 3 cages; 50.7 ± 19.7 (s.d.) | | | |
| | Hatch rate | Percentage of hatched eggs per iso-female; *w*Mel-infected female x *w*Mel-infected male; 50 females per 3 cages; 65 ± 26.9% (s.d.) | | | |
| | Cytoplasmic Incompatibility | Percentage of hatched eggs per iso-female; 50 females per 3 cages; uninfected female x *w*Mel-infected male; 0% | | | |
| | Maternal transmission | Percentage of offspring with *Wolbachia*; 50 females per 3 cages; 2 offspring per female; 98% | | | |
| | Insecticide mortality | Percentage of mosquito mortality when exposed to a given concentration of insecticide; three replicates of 14–25 mosquitoes; percentage mortality ± s.d. | | | |
| | | *w*Mel-COL | | WT1 | |
| | | Deltamethrin 0.05%– 100% | | Deltamethrin 0.05%– 100% | |
| | | Permethrin 0.75%– 100% | | Permethrin 0.75%– 94.3 ± 2% | |
| | | Bendiocarb 0.1%– 40.8 ± 36.1% | | Bendiocarb 0.1%– 34.9 ± 38.1% | |
| | | Malathion 5%– 100% | | Malathion 5%– 100% | |
| | Reduced vector competence | Percentage reduction in DENV; *w*Mel-infected vs uninfected; qPCR whole body DENV copies relative to *Rps17*; | | | |
| | | DENV Serotype 1–94.35 ± 2.4% (s.d.) | | | |
| | | DENV Serotype 2–99.94 ± 0.0% (s.d.) | | | |
| | | DENV Serotype 3–99.99 ± 0.0% (s.d.) | | | |
| | | DENV Serotype 4–99.99 ± 0.0% (s.d.) | | | |
| | | Additional testing has been published elsewhere [2,33]. | | | |
| *w*Mel-COL2 | Backcrossing source | Popular, Aranjuez, Doce de octubre, San Javier and Belén | | | |
| | Backcrossing method | Three generations of outcrossing with wild-derived males from different Medellín comunas for three generations, followed by two outcrossing with wildtype males selected by Permethrin 0.75% and finally crossed with wild-derived (from comunas with high resistance to permethrin) males at rates between 10–20%. Finally, *kdr* mutation selection was undertaken for two common alleles. | | | |
| | *Wolbachia* infection rate | Percentage of offspring with *w*Mel; *w*Mel-infected female x uninfected male; 100% | | | |
| | Fecundity | Eggs per iso-female; 50 females per 3 cages; 45.5 ± 22.7 (s.d.) | | | |
| | Hatch rate | Percentage of hatched eggs per iso-female; *w*Mel-infected female x *w*Mel-infected male; 50 females per 3 cages; 83.4 ± 25.9 (s.d.) | | | |
| | Cytoplasmic Incompatibility | Percentage of hatched eggs per iso-female; 50 females per 3 cages; uninfected female x *w*Mel-infected male; 0% | | | |
| | Maternal transmission | Percentage of offspring from iso-females infected with *Wolbachi*a; 47 *w*Mel-infected females mated with uninfected male; 5 offspring screened per female; 100% | | | |
| | Insecticide mortality | Three replicates of 25 mosquitoes; percentage mortality ± s.d. | | | |
| | | *w*Mel-COL2 | | Wild-derived Lines | |
| | | Permethrin 0.75% | 68 ± 2% | Permethrin 0.75% | Guayabal 69.2 ± 13.4%<br>Poblado 16 ± 7.4%<br>Doce de octubre 18.2 ± 16.3%<br>La América 18.2 ± 16.3% |

https://doi.org/10.1371/journal.pntd.0011642.t002

Vector competence was measured for *w*Mel-COL and wild-derived WT1 lines. Immature and adult mosquitoes were reared following Moreira et al [37]. Mosquito infection and DENV genomic quantification was undertaken according to Rancès et al. and Frentiu et al. [38,39]. DENV copy number was normalised against *Rps17*.

## Releases of *Wolbachia*-infected *Ae. aegypti*

*w*Mel-infected *Ae. aegypti* releases were targeted to residential and commercial areas. Areas deemed unsuitable for *Ae. aegypti*, such as uninhabited forested or vegetated areas, open or vacant areas, sporting fields, large industrial areas, and major transport infrastructure (major roads, highways) were generally excluded from releases. In some areas, security concerns or a lack of access to private property prevented deployment in all residential areas. The size ($km^2$) of each release area and the total size of each comuna were calculated, along with the residential population (S2 Table). Adult mosquito release rates were calculated by averaging the number of mosquitoes per release tube and multiplying this by the number of release tubes used in each area per week. For mosquito release containers (MRCs), release rates were determined by counting emerging mosquitoes from a subset of MRCs and multiplying this by the number deployed in each release area each week.

**Pilot releases in Paris.** Pilot releases were undertaken in the París comuna in Bello municipality in two phases over 18 months: Phase 1 releases throughout the central Paris neighbourhood area between June and December 2015, and Phase 2 releases throughout the rest of Paris comuna between June and August 2016 (Fig 1). The releases involved both adult and egg release methods, using the *w*Mel-COL release strain (see *Strain Development* methods above). For the adult releases, cups of 3–4 day old adult mosquitoes (approximately 150 mosquitoes per cup) were stacked in crates and transported from the insectary facility at Universidad de Antioquia to the release site by vehicle. For the Phase 1 Paris neighbourhood releases, the releases were undertaken on foot by members of a local community organisation Fundacion Mi Gente. Each week, volunteers from the local area met at a community centre and were provided cups of adult mosquitoes for releases. Each volunteer had a prescribed area where they would undertake releases. The volunteers released the mosquitoes from the street, between 5–6 hrs, each week. Each pair of volunteers released between 40–50 cups, with a total of 723 cups of mosquitoes released each week. Egg releases were undertaken by staff and involved the placement of MRCs in shaded locations throughout the community. The MRCs were white plastic polypropylene buckets with lids, with four 6 mm emergence holes drilled around the perimeter of the bucket, and a plastic lid. Containers were filled with 2 L of tap water and 2g of Tetramin, along with an egg strip containing approximately 200 eggs. Containers were placed in areas near the front boundary of houses. MRCs were replaced every 2–3 weeks. A total of 40 MRCs were set each week. For the Phase 2 releases in the remaining areas of the Paris comuna, the releases were undertaken as above, except that the adult mosquito releases were undertaken by staff. A total of 682 cups of mosquito were released per week for 10 weeks. MRC releases were undertaken as above, with 44 MRCs set each week for 10 weeks.

**Large-scale mosquito releases throughout Bello and Medellín.** Expanded releases throughout the remaining areas of Bello and in Medellín were phased over three years from late-2016 to mid-2019. Release cups containing approximately 150 mosquitoes per cup were stacked in crates and transferred to release sites in vehicles. In the release sites, the vehicles followed predetermined release routes with release locations at approximately 50 m intervals along publicly accessible roads. Overall, this equated to an average of 267 release locations per $km^2$ and varied between 130 releases per $km^2$ in Santa Ana and 377 releases per $km^2$ in Buenos Aires. At each release location, a staff member would open a release container by removing the mesh covering from the container which was extended outside of the window of the vehicle. The release cup was gently shaken and the adult mosquitoes were released from the cup. Once completed, the vehicle would proceed to the next release location. Each release vehicle contained approximately 700 release cups, with releases being undertaken over a 5–6 hr period.

Releases were undertaken between 7–13 hrs. Releases were undertaken weekly in each area for between 10–15 weeks in Phase 1 and 8–33 weeks in Phase 2.

For the Bello releases between October 2016 and November 2017, the Medellín case-control intervention areas (Aranjuez A, Manrique A and Santa Cruz) and Belén, El Poblado, Guayabal, Laureles-Estadio, Villa Hermosa releases between August 2017 and October 2017, the *w*Mel-COL release strain (see *Strain Development* methods above) was used. These releases were classified as phase one releases. In subsequent releases (hereby referred to as phase two releases), the *w*Mel-COL2 release line was used. These releases were undertaken between May 2018 and April 2019 in Bello and between October 2018 and October 2019 in Medellín. Release of *w*Mel-COL2 line *Wolbachia*-infected mosquitoes in the case-control intervention areas (Manrique A, Aranjuez A and Santa Cruz) began in August 2018 and concluded in March 2019. During this time supplementary egg releases were also undertaken in each of these areas. These involved placement of *Wolbachia*-infected *Ae*. *aegypti* (*w*Mel-COL2) egg strips or egg filled capsules, each containing approximately 100–150 eggs, into natural breeding sites. Approximately 1389, 843 and 1495 egg strips/capsules were distributed each week in Manrique A, Aranjuez A and Santa Cruz, respectively, over a four-week period (July–August 2018). Adult mosquito releases of *w*Mel-COL2 line in the previously untreated arms of the case-control study area (Aranjuez B, Manrique B and Popular) were undertaken between January and May 2022, after completion of the epidemiological study. Maps of phase 2 releases for Bello, Medellín, including the case control area, and Itagüí show the estimated number of mosquitoes released 100$m^2$ resolution (S2 –S7 Figs).

**Mosquito releases in Itagüí.** An initial phase (18 weeks) of *Wolbachia* mosquito releases were undertaken every one to two weeks between August 2019 and March 2020. These involved adult mosquito releases from a vehicle (555–590 release points per week over 11 weeks) in industrial areas, as described above for Bello and Medellín, and community-based egg releases in residential areas where participants from community groups and organisations volunteered to set up the MRCs around their homes. The community-based releases involved a specifically designed cardboard mosquito release container known as a Wolbicasa, with instructions for users on how to fill the container with water and add an egg capsule containing *Wolbachia* mosquito eggs. The volunteers then placed and maintained the MRCs outside their houses for 2–3 weeks. Between 57 and 1509 MRCs were distributed each week over 16 weeks. In late March 2020, all release and monitoring activities in Itagüí were stopped due to social distancing restrictions in response to the COVID-19 outbreak. In August 2020, social distancing restrictions were eased, and egg and adult releases recommenced. From August 2020 until November 2020, egg releases were undertaken by staff who distributed and set up Wolbicasas in public spaces throughout Itagüí (both industrial and residential areas). Releases were undertaken each week for 11 weeks, with between 1204 and 4324 Wolbicasas setup each week. From October 2020 until November 2020 adult mosquito releases were undertaken from motorcycles at 297–300 locations in industrial areas each week over 8 weeks. Finally, from November 2020 until December 2020 egg releases via MRCs were undertaken in public spaces at 1204–3032 locations in residential areas each week.

## Field monitoring

Mosquito collections were undertaken during and after releases using either BG Sentinel (BGS) traps (Biogents AG, Regensburg, Germany, Product number NR10030) or aspirator collections [20] (Improved Prokopack Aspirator Model 1419, John W. Hock Company, Gainesville, Florida, USA). BGS traps were placed in protected outdoor locations, near houses. Mosquitoes were collected from the BGS traps every 1–2 weeks. Aspirator collections were

undertaken inside houses over a 10–15 minute period, with operators visiting each accessible room and aspirating mosquitoes from resting locations (walls and behind curtains, under and behind furniture). Mosquito samples were returned to the laboratory for sorting, morphological identification and counting. *Aedes aegypti* samples were stored in 70% ethanol solution prior to screening for *Wolbachia* infection status.

During releases the density of collections (either BGS or aspirator collections) were approximately 16 per $km^2$ (one per 0.0625 $km^2$). Monitoring generally commenced within 1–4 weeks of releases and *Ae*. *aegypti* samples were screened for *Wolbachia* every month during releases. The sampling frequency varied due to logistical constraints and the large areas that were monitored (weekly numbers of BGS and aspirator collections peaked at 516 and 616, respectively). After completion of releases, mosquitoes were screened periodically, initially every month for up to 12 months after releases, then at 6–12 monthly intervals thereafter. Each month the *Wolbachia* infection frequency was calculated by dividing the number of samples that tested positive for *Wolbachia* by qPCR by the total number of samples tested. The sample sizes shown in Figs 2–5 represent the total number of samples tested per month (sample sizes varied due size of the reporting area and the number of collections each month, e.g., weekly, fortnightly or monthly collections.

Disruptions to field monitoring occurred between 16 March 2020 and 19 April 2021 due to social distancing restrictions in response to the COVID-19 outbreak, during which time no field collections were undertaken. In the Paris neighbourhood, post-release monitoring was undertaken for 5.1 years after releases were completed. In Bello and Medellín, post-release monitoring was undertaken for between 1.8–2.6 years after releases, except for the case-control study areas where monitoring was undertaken in three areas for 2.1–2.6 years after releases. No post-release monitoring was undertaken in the three control areas where releases were completed in April 2022.

## Training, data storage & mosquito population analysis

To ensure data integrity, WMP has developed customised web and mobile applications referred to as Core Data. Technologies used to develop the platform include Django, Python, Javascript and ODK-X applications.

The Core Data platform enables planning and completion of mosquito releases, and the collection of samples for *Wolbachia* measurement. An offline-enabled mobile app, with standardised data forms delivered on a map-based interface enables field data collection. A web-based field planning app allows field coordinators to develop release and monitoring plans on a map and manage the scheduling and assignment of field tasks to distributed field teams. Custom-built dashboards provide spatially enabled reporting of *Wolbachia* incidence results from individual traps and aggregated to reporting areas across the release site. The system was first implemented in Colombia in January 2017. Project implementation data captured prior to 2017 has been imported into the platform from spreadsheets.

Entomological data was exported from Core Data Entomological. Analysis of field and *w*Mel introgression data was conducted using R through RStudio. Data was collated and visualised using several R packages including 'ggplot2', 'tidyverse', 'lubridate' and 'ggh4x'. The data is available at 'https://doi.org/10.6084/m9.figshare.24045993.v1'.

# Results

## Pilot releases in París

Releases involving the *w*Mel-COL line were undertaken in the neighbourhood of París for 21 weeks between June–December 2015. Subsequent releases in the three surrounding

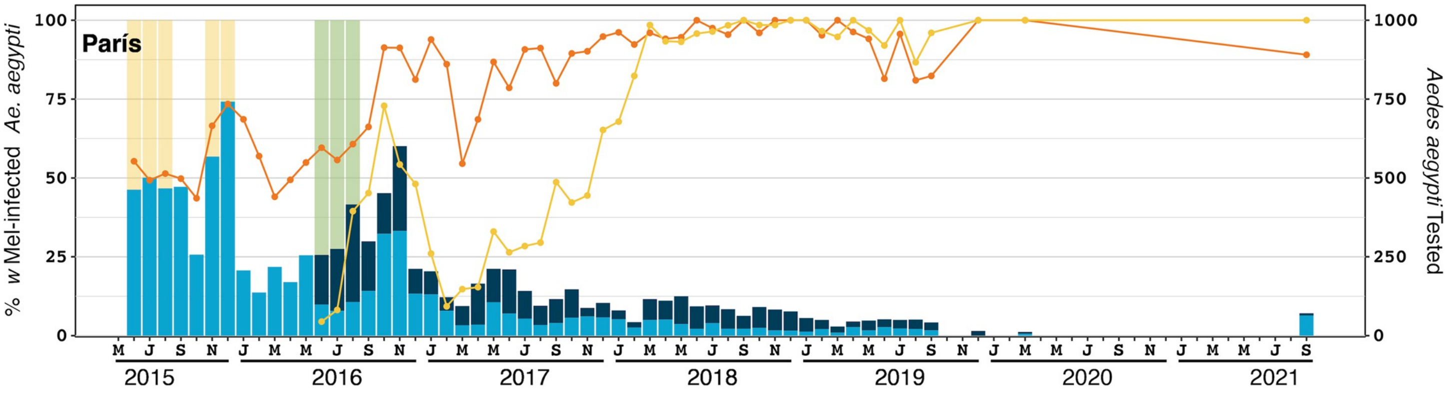


**Fig 2. *Wolbachia* establishment in París comuna, Colombia.** The lines (left axis) represent the percent of *Ae. aegypti* screened that were infected with *w*Mel *Wolbachia* in the initial release area in the París neighbourhood, shown with an orange line, and the wider París comuna excluding the initial release area, shown with a yellow line. Yellow shading indicates release periods in the París neighbourhood. Green shading indicates release periods in the wider París comuna. The stacked bars (right axis) indicate the number of *Ae. aegypti* screened within the París neighbourhood (light blue) and in the wider París comuna (dark blue). Monitoring events with less than five screened mosquitoes were omitted.

https://doi.org/10.1371/journal.pntd.0011642.g002

neighbourhoods (Los Sauces, Maruchenga and Nueva Jerusalem) in the París comuna were undertaken for 10 weeks between July–December 2016. Overall, an average of 12,460 *Wolbachia* mosquitoes were released per km$^2$ per week, across both release periods (S2 Table). In both the initial release and subsequent expansion, the *w*Mel infection frequency in mosquitoes was high at the end of the release period but declined shortly after (Fig 2). However, despite no additional releases in the surrounding areas in the Paris comuna, the *w*Mel frequency increased after this decline and has persisted in the local mosquito population, generally above 80%, for over 40 months (Fig 3).

## Bello and Medellín Phase 1 releases with the *w*Mel-COL line

Phase 1 releases involving the *w*Mel-COL line were undertaken in ten Bello comunas between October 2016 and November 2017. Both the duration of releases varied between 10–15 weeks and the numbers of *Wolbachia* mosquitoes released ranged between 12,352 and 41,563 per km$^2$/week (S2 Table). During the final month of releases, the prevalence of *Wolbachia* in field mosquitoes was high (60–80%) in only four areas (Altos de Niquia, Guasimalito, La Madera and Santa Anna), with the remaining areas found to have low *w*Mel infection prevalences of between 20–45%. Monitoring over the following 1–6 months found that the *w*Mel infection prevalence had decreased to less than 25% across all areas (Fig 3). Due to the failure of *w*Mel *Wolbachia* to persist in the mosquito populations after releases, despite the high prevalence of *Wolbachia* in field populations of mosquitoes during releases and the extended duration of these releases (generally between 10–15 weeks), no further releases of the *w*Mel-COL line were undertaken.

The six areas within the case control study were designated as intervention (Aranjuez A, Manrique A, Santa Cruz) or untreated (Aranjuez B, Manrique B, Popular). Releases into the three case control intervention comunas using the *w*Mel-COL strain were conducted from April to December 2017. The releases were undertaken over 15 weeks with between 37,975 and 39,259 mosquitoes per km$^2$/week (S2 Table). Only intermediate infection rates were found in mosquitoes at the completion of the releases (Aranjez A 55%, Manrique A 55%, Santa Cruz 45%). In each comuna, *w*Mel levels rapidly declined after releases ceased.

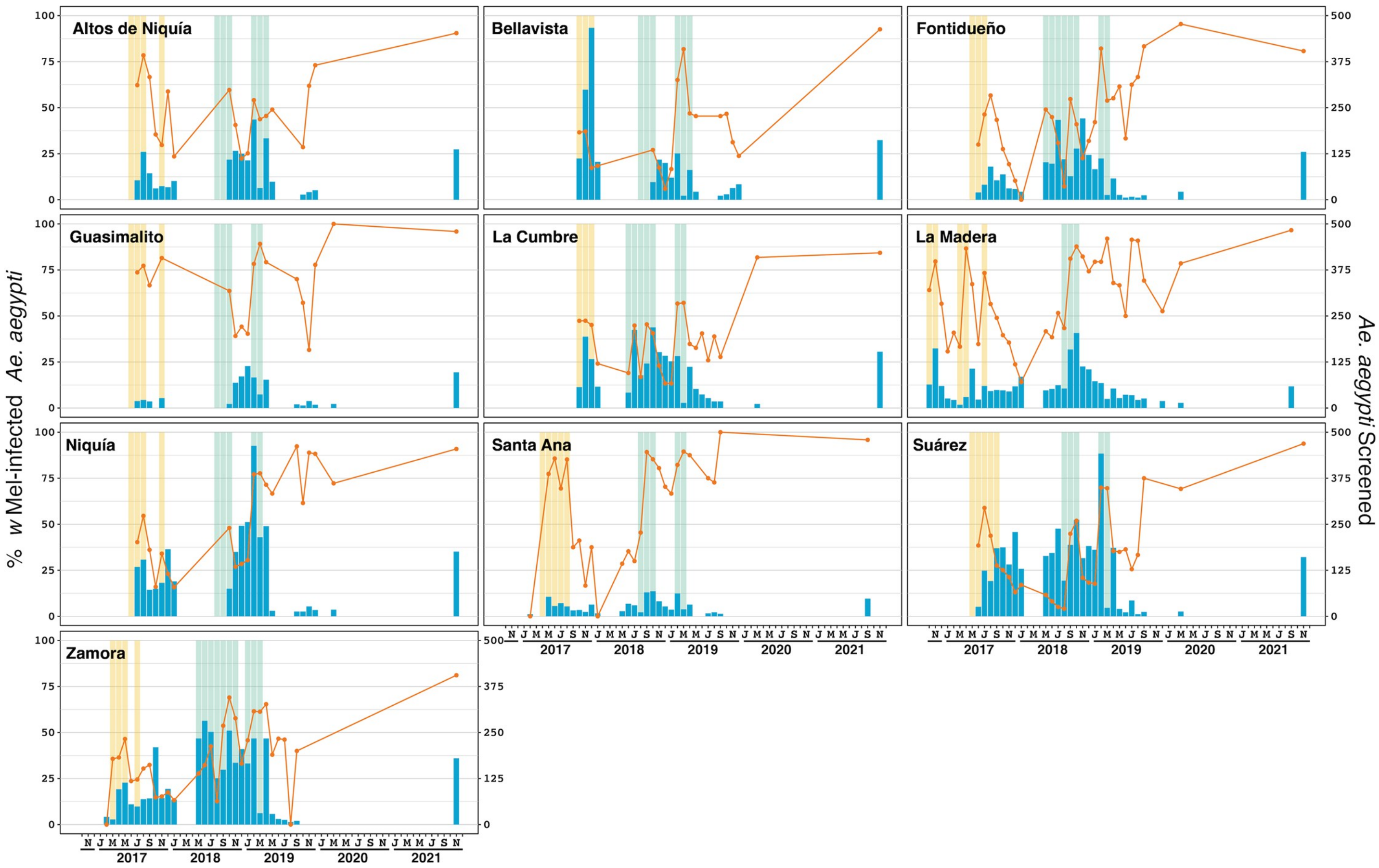


**Fig 3. *Wolbachia* infection prevalence over time in *Aedes aegypti* mosquitoes in ten deployment areas of Bello, Colombia.** The orange line (left axis) represents the percentage of *Ae. aegypti* tested that were infected with *w*Mel *Wolbachia*. Phase 1 releases, using the *w*Mel-COL line are shown with yellow shading. Phase 2 releases, using the *w*Mel-COL2 line, are shown with green shading. The blue bars (right axis) indicate the number of *Ae. aegypti* tested. Months with fewer than five *Ae. aegypti* tested have been omitted (n = 3 in Guasimalito; n = 2 in Santa Ana; n = 1 in La Cumbre).

https://doi.org/10.1371/journal.pntd.0011642.g003

## Bello and Medellín Phase 2 releases with the *w*Mel-COL2 line

The *w*Mel-COL2 (insecticide resistance matched; described in *w*Mel *Aedes aegypti* release lines above) *Wolbachia*-infected *Ae. aegypti* line was released throughout Bello from May 2018 to April 2019 (Fig 3 and S2 Table). Field monitoring activities were paused from April 2020 due to the COVID-19 pandemic, and recommenced in September-November 2021, after which time the *w*Mel infection frequency was found to be uniformly high across all Bello comunas (81.1 to 95.9%) (Table 3 and Fig 3).

Releases of the *w*Mel-COL2 *Wolbachia* mosquito line were undertaken across the 12 Medellín comunas between October 2018 and October 2019 (S2 Table). Compared to Bello, *Wolbachia* establishment in Medellín was highly variable (Fig 4 and Table 3). In comunas Buenos Aires, Castilla, Doce de Octubre, Guayabal, La Americas and La Candelaria *Wolbachia* infection frequencies were subsequently found to be high (76–98%) when monitoring recommenced in September to November 2021 (Fig 4 and Table 3). In El Poblado, San Javier and Villa Hermosa *Wolbachia* infection frequencies were found to be low (18–38%) when monitoring recommenced in July-September 2021 (Fig 4 and Table 3). In the three remaining comunas, Belen, Laureles-Estadio and Robledo the *Wolbachia* infection frequency was found to be at intermediate levels (33–56%) in September 2021 (Fig 4 and Table 3).

**Table 3. *Wolbachia* establishment in Bello & Medellín by comuna.**

| Comuna | Month of last *Wolbachia* release | Month of last *Wolbachia* monitoring | *w*Mel % at last monitoring |
|---|---|---|---|
| **Bello** | | | |
| Altos de Niquía | April 2019 | November 2021 | 90.5 |
| Bellavista | April 2019 | November 2021 | 92.6 |
| Fontidueño | March 2019 | November 2021 | 80.8 |
| Guasimalito | March 2019 | November 2021 | 95.9 |
| La Cumbre | March 2019 | November 2021 | 84.3 |
| La Madera | October 2018 | September 2021 | 96.6 |
| Niquía | March 2019 | November 2021 | 90.9 |
| Santa Ana | March 2019 | September 2021 | 95.8 |
| Suárez | March 2019 | November 2021 | 93.8 |
| Zamora | March 2019 | November 2021 | 81.1 |
| **Medellín** | | | |
| Belén | November 2019 | August 2021 | 32.5 |
| Buenos Aires | August 2019 | September 2021 | 86.6 |
| Castilla | October 2019 | September 2021 | 98.1 |
| Doce de Octubre | September 2019 | September 2021 | 89.2 |
| El Poblado | November 2019 | July 2021 | 18.4 |
| Guayabal | September 2019 | July 2021 | 76 |
| La América | October 2019 | August 2021 | 83 |
| La Candelaria | July 2019 | August 2021 | 92.3 |
| Laureles-Estadio | November 2019 | August 2021 | 56.4 |
| Robledo | September 2019 | September 2021 | 51.4 |
| San Javier | July 2019 | August 2021 | 24.2 |
| Villa Hermosa | July 2019 | September 2021 | 37.7 |
| **Case Control** | | | |
| Aranjuez A | May 2019 | January 2022 | 87.5 |
| Aranjuez B | April 2022 | April 2022 | 85.8 |
| Manrique A | March 2019 | January 2022 | 46.7 |
| Manrique B | April 2022 | April 2022 | 78.1 |
| Popular | April 2022 | April 2022 | 66 |
| Santa Cruz | March 2019 | December 2021 | 81.2 |

Final Percentage denotes percentage of *w*Mel infected *Aedes aegypti* caught in each comuna at the time of final monitoring.

https://doi.org/10.1371/journal.pntd.0011642.t003

There was no clear association between the number of releases and the outcome in terms of the level of *Wolbachia* establishment. The heterogeneity of the landscape and the fact that the releases of mosquitoes from vehicles were limited to publicly accessible roads, combined with the variable release period, meant that the overall cumulative dosing of released mosquitoes per km$^2$ varied significantly across the Medellín release areas. The cumulative release rates varied from 388,560/km$^2$ in El Poblado to 1,045,182/km$^2$ in Buenos Aires (average 769,458/km$^2$) (S2 Table). In Medellín there was a clear association between the cumulative numbers of mosquitoes released per km$^2$ and the overall *Wolbachia* infection frequency in mosquitoes measured between July and September 2021 (S8 Fig). The overall cumulative release numbers in each of the six areas where *Wolbachia* infection frequencies were high (76.0–92.3%) ranged from 746,611 to 1,045,182 mosquitoes per km$^2$ (average 942,152 mosquitoes per km$^2$). In comparison, overall cumulative release numbers in each of the six areas where *Wolbachia* infection frequencies were low (18.4–56.4%) ranged from 388,560 to 843,623 mosquitoes per km$^2$

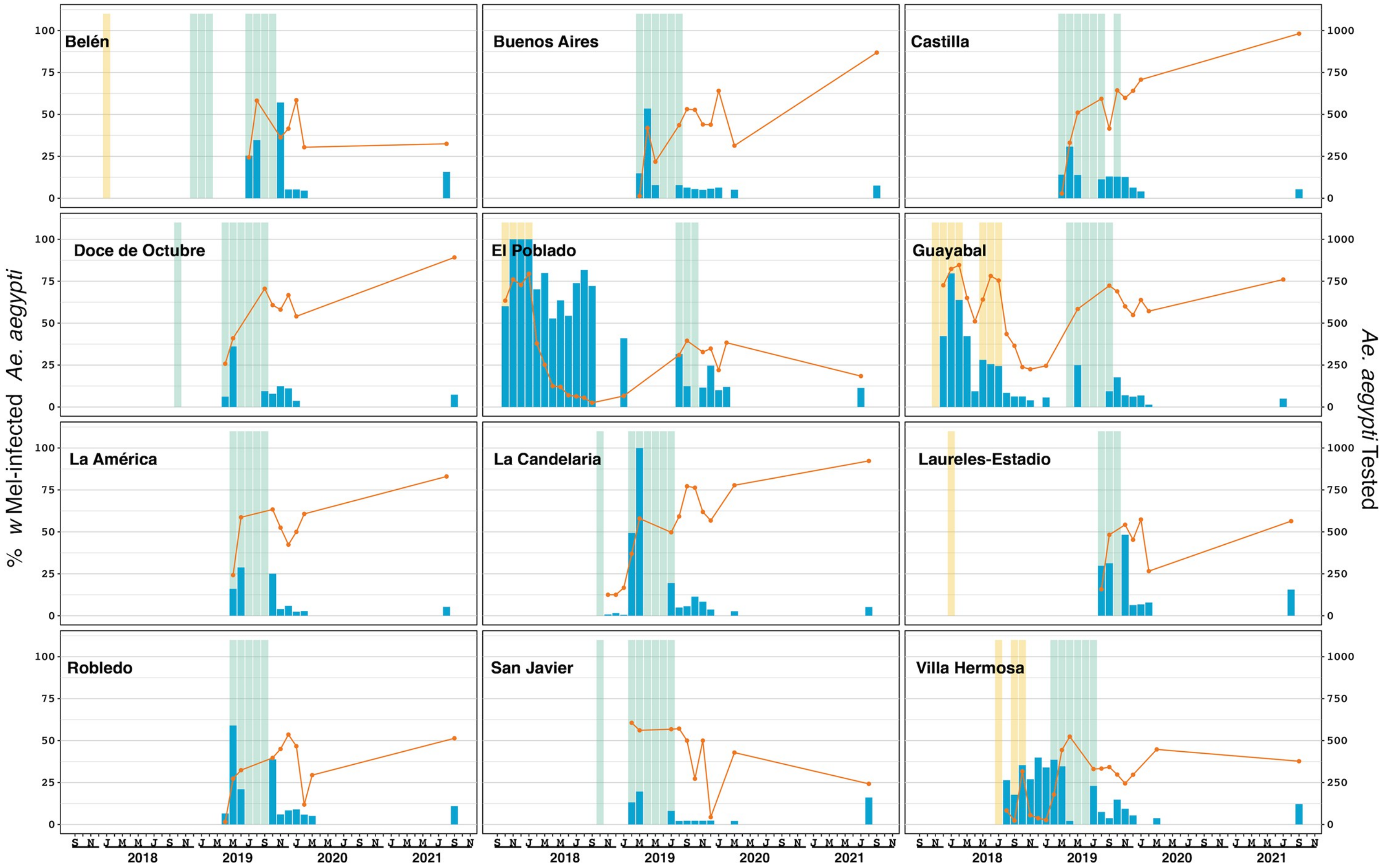


**Fig 4.** ***Wolbachia* infection prevalence over time in *Aedes aegypti* mosquitoes in 12 deployment areas of Medellín, Colombia.** The orange line (left axis) represents the percentage of *Ae. aegypti* tested that were infected with *w*Mel *Wolbachia*. Phase 1 releases using the *w*Mel-COL line are shown with yellow shading. Phase 2 releases, using the *w*Mel-COL2 line release periods are shown with orange and green shading. The blue bars (right axis) indicate the number of Ae. aegypti tested. To aid visualisation, months with greater than 1000 *Ae. aegypti* tested were capped at 1000 (n = 3 in Guayabal; n = 1 in La Candelaria).

https://doi.org/10.1371/journal.pntd.0011642.g004

(average 596,765 mosquitoes per km$^2$). To examine the heterogeneity in release numbers within the release areas, we mapped the cumulative numbers of mosquitoes released in 100 x 100m grid squares for each comuna. The release numbers varied within some sites, with significant areas having no releases or only limited numbers of mosquitoes released (S4 –S6 Figs). For example, in El Poblado, with 18.4% *Wolbachia* prevalence at last reading, 23.8% of the target release area had no releases undertaken due to the restricted access for the release vehicles. This area had high numbers of highrise buildings and gated apartment blocks which precluded access to release vehicles. This resulted in a patchwork effect, whereby a significant proportion of the area was underdosed in terms of release numbers. Similar patterns were found in Belen (14.2% no releases / 32% *Wolbachia* prevalence at last reading) and Robledo (19.0% no releases / 51.4% *Wolbachia* prevalence at last reading), which also had significant numbers of highrise buildings and gated apartment blocks. Other comunas that had high proportions of the areas without releases included Bella Vista 22.1%, Fontidueno 21.0%, Guasimalito 40.5%, Guayabal 24.2%, La Cumbre 22.8%, and Niquia 21.8%, despite achieving high levels of *Wolbachia* establishment. Overall, the landscape features in the release areas, particularly those with high numbers of highrise buildings and gated apartment blocks, may have contributed to poor *Wolbachia* establishment.

In the case control intervention areas, releases of the *w*Mel-COL2 line were undertaken from April 2018 to March 2019 (S2 Table). In addition to the release numbers in S2 Table, releases from egg strips and capsules placed in natural breeding sites in the case-control intervention areas occurred during a four-week period (July–August 2018). No estimates could be made of the adults emerging from these releases. Within Aranjuez A and Santa Cruz, *w*Mel-COL2 releases resulted in high *Wolbachia* prevalence (Fig 5). However, within Manrique A, the *Wolbachia* infection frequency has been highly variable (Fig 5). *Wolbachia* mosquito releases in the untreated case-control areas (Aranjuez B, Manrique B and Popular) were undertaken between February and April 2022, with *Wolbachia* prevalence ranging from 60–80% at the completion of releases.

## Itagüí deployment

Itagüí mosquito releases involving the *w*Mel-COL2 mosquito line were undertaken between August 2019 and November 2020, and incorporated both adult mosquito releases, and eggs releases via both community and staff-based methods. Combined adult, community egg and

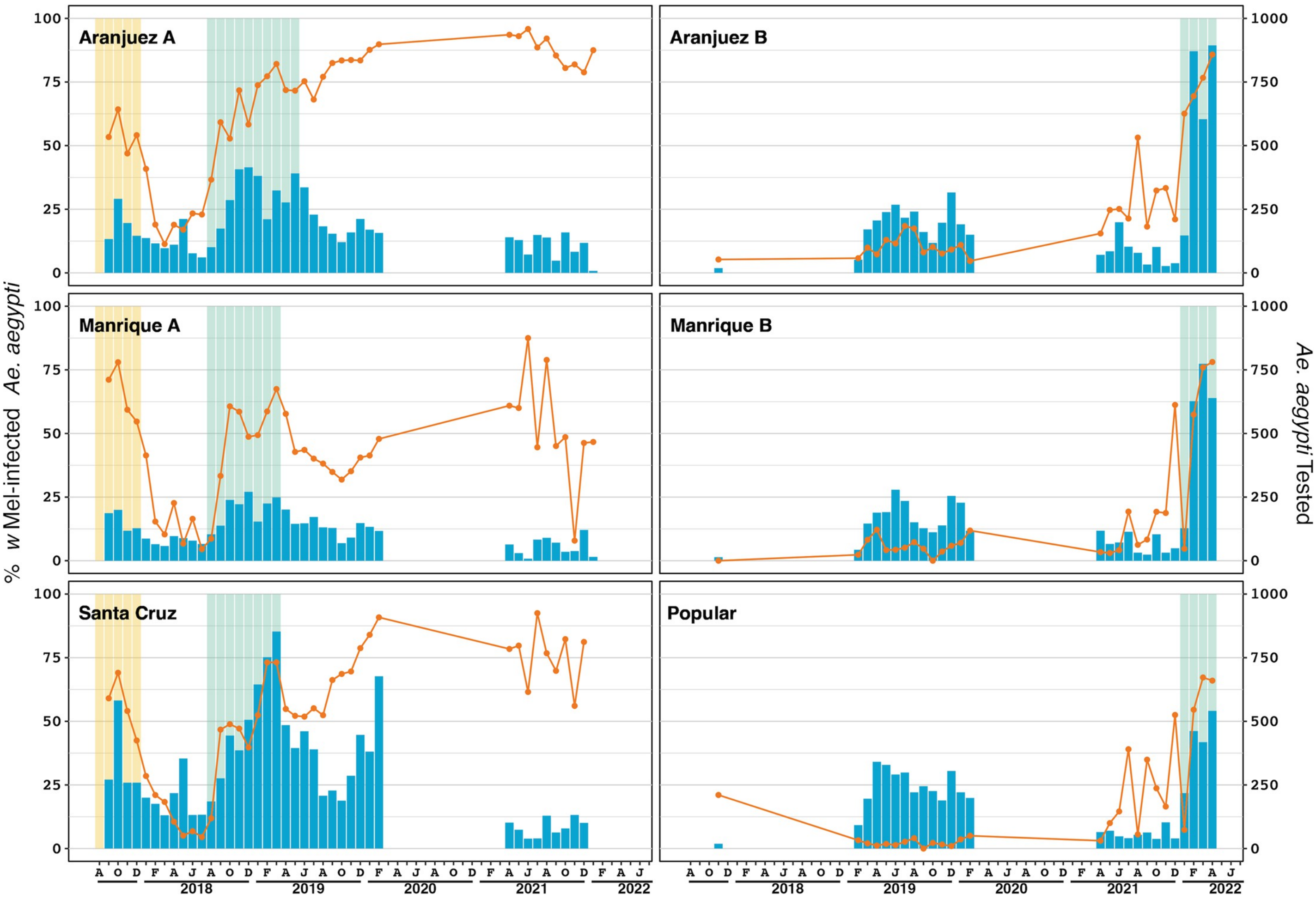


**Fig 5. *Wolbachia* introgression in the Medellín case control study, in the Aburrá Valley, Colombia.** The orange line (left axis) represents the percent of *Ae. aegypti* screened that were infected with *w*Mel *Wolbachia*. Phase one releases, using the *w*Mel-COL line, are shown with yellow shading. Phase two releases, using the *w*Mel-COL2 line are shown with green shading. The blue bars (right axis) indicate the number of *Ae. aegypti* screened. Months with fewer than five *Ae. aegypti* tested have been omitted (n = 1 in Popular; n = 1 in Santa Cruz).

https://doi.org/10.1371/journal.pntd.0011642.g005

MRC releases were undertaken between August and March 2020 (S2 Table). In late March 2020, all release and monitoring activities in Itagüí were stopped due to social distancing restrictions in response to the COVID-19 outbreak. In September 2020, egg and adult mosquito releases recommenced and were undertaken for 8 and 14 weeks, respectively (S2 Table). *Wolbachia* monitoring was not commenced in Itagüí until February 2020, and was paused from April 2020 due to COVID-19 restrictions. At the completion of releases the *Wolbachia* infection frequency in mosquitoes was 90% (Fig 6). Periodic monitoring in April and November 2021 found that the *Wolbachia* infection frequency in mosquitoes has remained high at 63.6 and 92.3%, respectively (Fig 6).

## Discussion

We have detailed the release of *w*Mel *Wolbachia* mosquitoes across the complex urban settings of Medellín, Bello and Itagüí, Colombia. Together these comprise 3.3 million people living in an area of 135 $km^2$. To our knowledge, this represents the largest single implementation of any method involving the release of mosquitoes in the world. This includes *Wolbachia* replacement methods that aim to introduce *Wolbachia* into mosquito populations to reduce pathogen transmission [6,8,10–17,25,40,41], or a range of different suppression methods involving sterile insect technique [42], incompatible insect technique [43–45] and combinations thereof [46–48], and transgenically modified mosquitoes containing a dominant lethal gene [49,50], that aim to reduce the size of the mosquito population.

Large scale community and stakeholder engagement and communication campaigns [11,12,24] were successful in raising broad awareness and high levels of acceptance for releases (S1 Table). Most community members were familiar with the conventional control methods including source reduction and application of insecticides to suppress mosquito abundance. Messaging to the communities emphasised the continuance of these preventative methods in parallel with *Wolbachia* mosquito releases. In the city of Medellín, the Health Secretariat complemented the National Dengue Control Program by adding spraying with insecticides when there was an increase in the density of mosquitoes. This was measured using ovitraps and identification of mosquitoes with arboviruses in the entomo-virological surveillance [51]. In the cities of Bello and Itagüí, the National Dengue Control Program was maintained, which only recommends the spraying of insecticides when there are outbreaks of the disease. During the reporting period, no outbreaks occurred in any of the three cities. Interestingly, in Medellín it

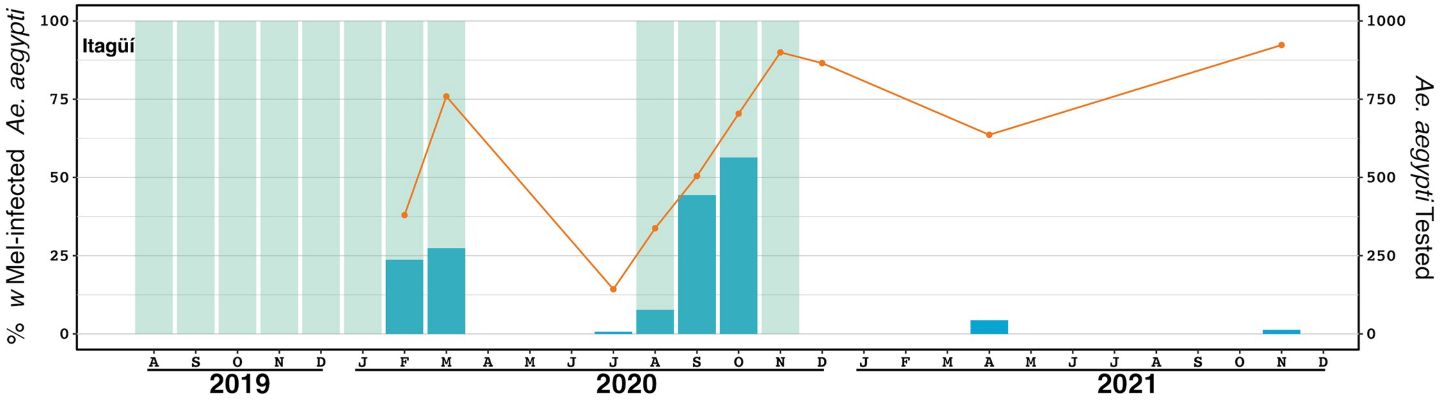


**Fig 6. *Wolbachia* introgression in Itagüí, Colombia.** The orange line (left axis) represents the percent of *Ae. aegypti* screened that were infected with *w*Mel *Wolbachia*. Green shading indicates release periods using the *w*Mel-COL2 line. The blue bars (right axis) indicate the number of *Ae. aegypti* screened. Monitoring events with less than five screened mosquitoes are omitted.

https://doi.org/10.1371/journal.pntd.0011642.g006

was noted that during 2021, the number of properties treated with insecticides was 17 times lower compared to 2020, due to operational issues and the low circulation of arboviruses in mosquitoes and the low incidence of dengue [51]. It is anticipated that *Wolbachia* establishment may reduce the frequency of reactive insecticide spraying in response to local disease transmission as was found in Yogyakarta, Indonesia [52].

The variable outcomes in terms of the *w*Mel *Wolbachia* mosquito releases in the current study are in contrast to the previous releases of *w*Mel in North Queensland and Indonesia that showed a relatively constant and rapid increase of *Wolbachia* infection in mosquitoes after approximately 12 rounds of releases (12–24 weeks depending on weekly or fortnightly release cycles) [10–12,14], followed by establishment of *w*Mel at a high level and its persistence in the local mosquito population within 6 months of completion of releases. The initial pilot release in the Paris neighbourhood in Bello was typical of these previous releases, and although there was an initial drop in *w*Mel infection frequency in the Paris neighbourhood after completion of releases, the frequency recovered and reached >90% within 10 months of completion of releases.

For the subsequent expanded Paris comuna and Bello and Medellín Phase 1 releases, we observed a trend of medium to high *w*Mel infection frequencies in mosquitoes during the release periods (10–15 weeks); however, there was a rapid decline in the *w*Mel infection prevalence in mosquitoes, across all areas, over the proceeding 6 months. This trend was similar to that observed in the initial small-scale releases in Tubiacanga, Brazil [5] that involved releases of a *w*Mel mosquito line with low insecticide resistance, compared with wild caught mosquitoes that were subsequently found to have high resistance to pyrethroid insecticides. In Tubiacanga, re-releases involving *w*Mel on a pyrethroid resistant mosquito background that was matched to the local mosquito population, resulted in high *w*Mel *Wolbachia* frequency after 18 weeks of releases, and the frequency of *Wolbachia* remained at 85–90% one year after releases [5]. In the Colombian releases reported here, we similarly conclude that the improved matching of the pyrethroid resistance profile of the *w*Mel release material with the field populations of mosquitoes resulted in local *Wolbachia* establishment. Given pyrethroid-resistance is widely distributed in *Ae. aegypti* populations, future *Wolbachia* deployments should pay attention to maintaining insecticide resistance in release lines.

*Wolbachia* infection frequencies remained intermediate (<60%) at the time of last monitoring in seven of 30 release areas, all of which are in Medellín. Various factors may have contributed to this variability, including the lower overall cumulative mosquito releases numbers in these areas (S2 Table and S8 Fig) and the heterogeneity in the landscape features which limited the access of release vehicles. For the areas in Medellín where *Wolbachia* was established in the mosquito populations, releases occurred for between 20–31 weeks and with release rates of between 30,381 to 52,259 mosquitoes per km$^2$ per week. These release data provide some guidance to future operational programs on the required duration and density of releases.

Large scale releases of *w*Mel *Wolbachia* in areas of Rio de Janeiro, Brazil, reported that *Wolbachia* establishment was variable in some areas, with moderate *Wolbachia* prevalence during releases (30–60%), but not self-sustaining in mosquito populations after the completion of releases [7]. The heterogeneity in *Wolbachia* establishment was thought to be due to the complex urban settings, including significant spatial variation in the baseline *Ae. aegypti* populations and limited access to some areas, such as favela communities. Mathematical modelling studies predict that the spread and establishment of *Wolbachia* will be slower in landscapes with high spatio-temporal variation in mosquito demographics and key environmental parameters [53]. Given the large scale of the releases across Medellín, it is likely that there were similar small-scale heterogeneities in *Ae. aegypti* abundance or unknown environmental factors related to *Wolbachia* establishment. Improved release strategies, involving the tailored and

supplementary releases of mosquitoes into niche habitats that can't be reached by vehicle-based releases of adults from public roads, may facilitate more uniform *Wolbachia* establishment.

The average temperatures in Medellín and Bello (average daily temperatures between 21.8–23.1˚C) were relatively mild compared with other areas where *Wolbachia* has been successfully implemented (Australia 21.7–27.9˚C; Indonesia 25.4–27.0˚C). At an average temperature of 22˚C *Ae. aegypti* immature development times, including embryonic development, and larval and pupal stages are approximately double (21–25 days) those observed at 28˚C (12–13 days) [54]. Together with an extended gonotrophic cycle of 8 days at 20˚C, compared with 2–3 days at 26–30˚C [55], means that the generational turnover of *Ae. aegypti* is likely to exceed 4–5 weeks in Medellín, compared with 2–3 weeks at warmer temperatures. The extended mosquito development times in Medellín may result in slower *Wolbachia* establishment. Ongoing monitoring in areas with intermediate *Wolbachia* infection frequencies (Belen, El Poblado, Laureles-Estadio, Robledo, San Javier, Villa Hermosa) will determine whether *w*Mel *Wolbachia* becomes established in the local mosquito populations.

Despite the challenges due to interruptions to *Wolbachia* mosquito release and monitoring activities due to COVID-19, there are several key lessons that may lead to improved operational outcomes from future *Wolbachia* mosquito releases. First, attention needs to be paid to understanding the insecticide resistance profile of the local mosquito populations and matching the profile of released mosquitoes to the local mosquitoes. Despite the initial establishment of *Wolbachia* in mosquitoes in the Paris releases, the subsequent Phase 1 releases in Bello and Medellín were unsuccessful due the lower pyrethroid-resistance levels in the released mosquitoes, compared with the local wild-type mosquitoes. Given that pyrethroid-resistance levels are widespread in *Ae. aegypti*, attention should be paid to maintaining insecticide resistance in release lines. This should include a pre-release study of local *Ae. aegypti* populations to facilitate identification of insecticide resistance profiles, along with periodic checks of released mosquitoes to ensure equivalence. Second, careful consideration needs to be given to the planning and design of mosquito release activities to ensure adequate dosage of released mosquitoes. In Medellín, some areas contained large numbers of high rise buildings and gated communities which were not suited to the release of mosquitoes from public roadways. These areas may require a more tailored release strategy to ensure adequate coverage. In Selangor State Malaysia, *Wolbachia* mosquito releases were undertaken in high density urban settings containing medium storey flats (up to 5 floors) and high rise apartments (up to 18 floors) [41]. In these settings, releases were undertaken on a grid basis within flats on the first and second floors, and on a grid basis on every third floor within the high rise apartments. Releases continued until *Wolbachia* frequencies exceeded 90%, with intensive monitoring of mosquito population across different floors of the flats and apartments using ovitraps [41]. *Wolbachia* establishment was high in these areas, but required high density releases inside the high rise apartments and flats and intensive monitoring [41]. Based on the low *Wolbachia* establishment in similar areas in Medellín, releases may need optimisation to ensure adequate mosquito dosing. This may require manual releases of mosquitoes throughout apartment blocks and gated communities. This will likely require the release of higher numbers of mosquitoes in or around these areas and a tailored engagement strategy with apartment occupiers and administrators to facilitate access to these buildings. Third, the scale of the releases in the current study (coverage of 135 $km^2$) necessitated a reduced monitoring intensity compared with releases that have been undertaken previously [11,12]. Following the declaration of Zika as a public health emergency by the WHO and the guidelines/recommendations outlined within this declaration [18], the pace of *Wolbachia* deployment was greatly increased. This necessitated larger release areas, particularly in Medellín, which resulted in large monitoring areas and area wide estimates of

*Wolbachia* infection. However, we recognise that heterogeneities in local mosquito abundance and landscape features affect *Wolbachia* establishment that would not be identified using low-density monitoring. Future monitoring of large-scale operational deployments of *Wolbachia* may therefore need to be optimised to suit the local setting, perhaps based on prioritisation of representative areas for more intensive and frequent monitoring, as opposed to periodic monitoring across all release areas. This may provide more timely feedback on *Wolbachia* release performance and provide guidance for additional mosquito releases in areas where *Wolbachia* establishment is progressing slowly or is unlikely to become established.

While describing the operational complexities and disrupted monitoring of large-scale *w*Mel *Wolbachia* mosquitoes releases across a large heterogeneous urban area, we remain optimistic that over 3.3 million residents have been afforded long-term protection of *Wolbachia*. Generally, within six months of release completion, *Wolbachia* was stable and established at consistent levels (>60% prevalence) in the majority (67%) of the release areas. However, ongoing monitoring in these areas will determine whether *Wolbachia* persists and whether it establishes in the remaining areas. The impact of these *w*Mel *Wolbachia* deployment on the reduction of the incidence of notified dengue cases and virologically-confirmed dengue is described in Velez et al. [56]. Therein, using an interrupted time series analysis, the incidence of dengue was shown to be reduced by 95% in Bello, 94% in Medellín and 97% in Itagüí, following establishment of *w*Mel at ≥60% prevalence, compared to the pre-intervention period and after adjusting for seasonal trends. However, low patient enrolment in the case control study complicated analysis for this aspect of the work.

## Supporting information

**S1 Table. Engagement Activities.** Participation activities are those where members of the community directly interact with WMP staff and partners. Communication activities are semi-targeted advertising.
(DOCX)

**S2 Table. *Wolbachia*-infected *Aedes aegypti* Mosquito Release Numbers.**
(DOCX)

**S1 Fig. Medellín Climate Data January 2016—April 2023.** The average daily temperature per month is indicated in dark blue. The cumulative monthly rainfall (mm) is indicated in light blue. Data is derived from the weather station located at the Medellín Olaya Herrera Airport and extracted from the National Climate Data Centre (USA). Precipitation data from 2020 to 2021 was absent from the available data set.
(TIF)

**S2 Fig. Phase 2 release & monitoring of *w*Mel-infected *Aedes aegypti* within six comuna of Bello in the Aburrá Valley, Colombia.** Each comuna was divided into a $100m^2$ grid with grid squares lacking mosquito releases omitted (maps produced in QGIS version 3.16.1 using administrative boundaries for the municipal government of Bello (https://www.datos.gov.co/Ordenamiento-Territorial/Divisi-n-Pol-tico-Administrativa-Barrios-Bello-Ant/pnhh-ccwd) and OpenMapTiles basemap layer (https://openmaptiles.org/) with CARTO light design (https://carto.com/)). Release gradient was determined by using GPS coordinates of each release event and assigning the number of *w*Mel-infected mosquitoes to a corresponding grid square. Monitoring numbers were determined in the same way.
(TIF)

**S3 Fig. Phase 2 release & monitoring of *w*Mel-infected *Aedes aegypti* within four comuna of Bello in the Aburrá Valley, Colombia.** Each comuna was divided into a 100m$^2$ grid with grid squares lacking mosquito releases omitted (maps produced in QGIS version 3.16.1 using administrative boundaries for the municipal government of Bello (https://www.datos.gov.co/Ordenamiento-Territorial/Divisi-n-Pol-tico-Administrativa-Barrios-Bello-Ant/pnhh-ccwd) and OpenMapTiles basemap layer (https://openmaptiles.org/) with CARTO light design (https://carto.com/)). Release gradient was determined by using GPS coordinates of each release event and assigning the number of *w*Mel-infected mosquitoes to a corresponding grid square. Monitoring numbers were determined in the same way.
(TIF)

**S4 Fig. Phase 2 release & monitoring of *w*Mel-infected *Aedes aegypti* within six comuna of Medellín in the Aburrá Valley, Colombia.** Each comuna was divided into a 100m$^2$ grid with grid squares lacking mosquito releases omitted (maps produced in QGIS version 3.16.1 using administrative boundaries for the municipal government of Medellín (https://data.metabolismofcities.org/library/maps/35283/view/) and OpenMapTiles basemap layer (https://openmaptiles.org/) with CARTO light design (https://carto.com/)). Release gradient was determined by using GPS coordinates of each release event and assigning the number of *w*Mel-infected mosquitoes to a corresponding grid square. Monitoring numbers were determined in the same way.
(TIF)

**S5 Fig. Phase 2 release & monitoring of *w*Mel-infected *Aedes aegypti* within 6 comuna of Medellín in the Aburrá Valley, Colombia.** Each comuna was divided into a 100m$^2$ grid with grid squares lacking mosquito releases omitted (maps produced in QGIS version 3.16.1 using administrative boundaries for the municipal government of Medellín (https://data.metabolismofcities.org/library/maps/35283/view/) and OpenMapTiles basemap layer (https://openmaptiles.org/) with CARTO light design (https://carto.com/)). Release gradient was determined by using GPS coordinates of each release event and assigning the number of *w*Mel-infected mosquitoes to a corresponding grid square. Monitoring numbers were determined in the same way.
(TIF)

**S6 Fig. Phase 2 release & monitoring of *w*Mel-infected *Aedes aegypti* within the 6 Medellín case control areas in the Aburrá Valley, Colombia.** Each comuna was divided into a 100m$^2$ grid with grid squares lacking mosquito releases omitted (maps produced in QGIS version 3.16.1 using administrative boundaries for the municipal government of Medellín (https://data.metabolismofcities.org/library/maps/35283/view/) and OpenMapTiles basemap layer (https://openmaptiles.org/) with CARTO light design (https://carto.com/)). Release gradient was determined by using GPS coordinates of each release event and assigning the number of *w*Mel-infected mosquitoes to a corresponding grid square. Monitoring numbers were determined in the same way.
(TIF)

**S7 Fig. Phase 2 release & monitoring of *w*Mel-infected *Aedes aegypti* within Itagüí in the Aburrá Valley, Colombia.** The area was divided into a 100m$^2$ grid with grid squares lacking mosquito releases omitted (map produced in QGIS version 3.16.1 using administrative for the municipal government of Itagüí (https://www.datos.gov.co/Ordenamiento-Territorial/Localizaci-n-Geogr-fica-de-los-Barrios-del-Municip/didi-drqa)) and OpenMapTiles basemap layer (https://openmaptiles.org/) with CARTO light design (https://carto.com/)). Release gradient was determined by using GPS coordinates of each release event and assigning the

number of *w*Mel-infected mosquitoes to a corresponding grid square. Monitoring numbers were determined in the same way.
(TIF)

**S8 Fig. Relationship between *w*Mel prevalence monitoring & phase 2 releases per km$^2$ of *w*Mel-infected *Aedes aegypti*.** The predicted line from a linear model fit between prevalence of *w*Mel at time of last monitoring and the number of *w*Mel-infected mosquitoes released per km$^2$ in a given area. Values are provided in S2 Table.
(TIF)

## Acknowledgments

We would like to thank all members of the World Mosquito Program, our partners and the Aburrá Valley community for their involvement and support for the project. In particular we would like to thank the following people: Adriana Gaviria, Andrea Trujillo, Gustavo Blandon, Juanita Puchulu, Juliana Madrigal, Katherine Caviedes, Lina Zuluaga, Lorena Espinal, Sandra Naranjo, Santiago Zapata, Sebastian Perez, Veronica Jaramillo and Victor Villa.

## Author Contributions

**Conceptualization:** Iván Darío Velez, Peter A. Ryan, Scott L. O'Neill.

**Data curation:** Alexander Uribe, Luis Martínez, Benjamin R. Green, Tibor Frossard, Jack Brown-Kenyon, Nelson Grisales, Jai A. Denton.

**Formal analysis:** Jai A. Denton, Katherine L. Anders, Peter A. Ryan, Scott L. O'Neill.

**Investigation:** Iván Darío Velez, Alexander Uribe, Jovany Barajas, Sandra Uribe, Sandra Ángel, Juan David Suaza-Vasco, Maria Camila Mejia Torres, María Patricia Arbeláez, Eduardo Santacruz-Sanmartin, Lorena Duque, Tania Posada, Ana Cristina Patiño, Sandra Milena Gonzalez, Ana Lucía Velez, Jennifer Ramírez, Marlene Salazar, Sandra Gómez, Jorge E. Osorio, Inaki Iturbe-Ormaetxe, Yi Dong, Frederico C. Muzzi, Edwige Rances, Petrina H. Johnson, Ruth Smithyman, Bruno Col, D. Albert Joubert, Nelson Grisales, Scott A. Ritchie, Jai A. Denton, Jeremie R. L. Gilles, Katherine L. Anders, Simon C. Kutcher, Peter A. Ryan, Scott L. O'Neill.

**Methodology:** Iván Darío Velez, Katherine L. Anders, Peter A. Ryan, Scott L. O'Neill.

**Project administration:** Iván Darío Velez, Simon C. Kutcher.

**Resources:** Iván Darío Velez, Scott L. O'Neill.

**Supervision:** Iván Darío Velez, Scott L. O'Neill.

**Visualization:** Tibor Frossard, Jai A. Denton.

**Writing – original draft:** Iván Darío Velez, Jai A. Denton, Katherine L. Anders, Peter A. Ryan.

**Writing – review & editing:** Alexander Uribe, Nelson Grisales, Scott A. Ritchie, Jeremie R. L. Gilles, Simon C. Kutcher, Scott L. O'Neill.

## References

1. Walker T, Johnson PH, Moreira LA, Iturbe-Ormaetxe I, Frentiu FD, McMeniman CJ, et al. The *w*Mel *Wolbachia* strain blocks dengue and invades caged *Aedes aegypti* populations. Nature. 2011; 476: 450–453. https://doi.org/10.1038/nature10355 PMID: 21866159
2. Aliota MT, Walker EC, Yepes AU, Velez ID, Christensen BM, Osorio JE. The *w*Mel Strain of *Wolbachia* Reduces Transmission of Chikungunya Virus in *Aedes aegypti*. PLoS Negl Trop Dis. 2016; 10: e0004677. https://doi.org/10.1371/journal.pntd.0004677 PMID: 27124663
3. Flores HA, Bruyne JT de, O'Donnell TB, Nhu VT, Giang NT, Trang HTX, et al. Multiple *Wolbachia* strains provide comparative levels of protection against dengue virus infection in *Aedes aegypti*. PLoS Pathog. 2020; 16: e1008433. https://doi.org/10.1371/journal.ppat.1008433 PMID: 32282862
4. Pocquet N, O'Connor O, Flores HA, Tutagata J, Pol M, Hooker DJ, et al. Assessment of fitness and vector competence of a New Caledonia *w*Mel *Aedes aegypti* strain before field-release. PLoS Negl Trop Dis. 2021; 15: e0009752. https://doi.org/10.1371/journal.pntd.0009752 PMID: 34492017
5. Garcia G de A, Sylvestre G, Aguiar R, Costa GB da, Martins AJ, Lima JBP, et al. Matching the genetics of released and local *Aedes aegypti* populations is critical to assure *Wolbachia* invasion. PLoS Negl Trop Dis. 2019; 13: e0007023. https://doi.org/10.1371/journal.pntd.0007023 PMID: 30620733
6. Gesto JSM, Ribeiro GS, Rocha MN, Dias FBS, Peixoto J, Carvalho FD, et al. Reduced competence to arboviruses following the sustainable invasion of *Wolbachia* into native *Aedes aegypti* from Southeastern Brazil. Sci Rep. 2021;11. https://doi.org/10.1038/s41598-021-89409-8 PMID: 33976301
7. Gesto JSM, Pinto SB, Dias FBS, Peixoto J, Costa G, Kutcher S, et al. Large-Scale Deployment and Establishment of *Wolbachia* Into the *Aedes aegypti* Population in Rio de Janeiro, Brazil. Front Microbiol. 2021;12. Available: https://doi.org/10.3389/fmicb.2021.711107 PMID: 34394061
8. Hoffmann AA, Montgomery BL, Popovici J, Iturbe-Ormaetxe I, Johnson PH, Muzzi F, et al. Successful establishment of *Wolbachia* in *Aedes* populations to suppress dengue transmission. Nature. 2011; 476: 454–457. https://doi.org/10.1038/nature10356 PMID: 21866160
9. Hoffmann AA, Iturbe-Ormaetxe I, Callahan AG, Phillips BL, Billington K, Axford JK, et al. Stability of the *w*Mel *Wolbachia* Infection following Invasion into *Aedes aegypti* Populations. PLoS Negl Trop Dis. 2014; 8: e3115. https://doi.org/10.1371/journal.pntd.0003115 PMID: 25211492
10. Indriani C, Tantowijoyo W, Rancès E, Andari B, Prabowo E, Yusdi D, et al. Reduced dengue incidence following deployments of *Wolbachia*-infected *Aedes aegypti* in Yogyakarta, Indonesia: a quasi-experimental trial using controlled interrupted time series analysis. Gates Open Res. 2020; 4: 50. https://doi.org/10.12688/gatesopenres.13122.1 PMID: 32803130
11. O'Neill SL, Ryan PA, Turley AP, Wilson G, Retzki K, Iturbe-Ormaetxe I, et al. Scaled deployment of *Wolbachia* to protect the community from dengue and other *Aedes* transmitted arboviruses. Gates Open Res. 2019; 2: 36. https://doi.org/10.12688/gatesopenres.12844.2 PMID: 30596205
12. Ryan PA, Turley AP, Wilson G, Hurst TP, Retzki K, Brown-Kenyon J, et al. Establishment of *w*Mel *Wolbachia* in *Aedes aegypti* mosquitoes and reduction of local dengue transmission in Cairns and surrounding locations in northern Queensland, Australia. Gates Open Res. 2020; 3: 1547. https://doi.org/10.12688/gatesopenres.13061.2 PMID: 31667465
13. Tantowijoyo W, Andari B, Arguni E, Budiwati N, Nurhayati I, Fitriana I, et al. Stable establishment of *wMel Wolbachia* in *Aedes aegypti* populations in Yogyakarta, Indonesia. PLoS Negl Trop Dis. 2020; 14: e0008157. https://doi.org/10.1371/journal.pntd.0008157 PMID: 32302295
14. Utarini A, Indriani C, Ahmad RA, Tantowijoyo W, Arguni E, Ansari MR, et al. Efficacy of *Wolbachia*-Infected Mosquito Deployments for the Control of Dengue. N Engl J Med. 2021; 384: 2177–2186. https://doi.org/10.1056/NEJMoa2030243 PMID: 34107180
15. Schmidt TL, Barton NH, Rašić G, Turley AP, Montgomery BL, Iturbe-Ormaetxe I, et al. Local introduction and heterogeneous spatial spread of dengue-suppressing *Wolbachia* through an urban population of *Aedes aegypti*. PLoS Biol. 2017; 15: e2001894. https://doi.org/10.1371/journal.pbio.2001894 PMID: 28557993
16. Pinto SB, Riback TIS, Sylvestre G, Costa G, Peixoto J, Dias FBS, et al. Effectiveness of *Wolbachia*-infected mosquito deployments in reducing the incidence of dengue and other Aedes-borne diseases in Niterói, Brazil: A quasi-experimental study. PLoS Negl Trop Dis. 2021; 15: e0009556. https://doi.org/10.1371/journal.pntd.0009556 PMID: 34252106
17. dos Santos GR, Durovni B, Saraceni V, Riback TIS, Pinto SB, Anders KL, et al. Estimating the effect of the *w*Mel release programme on the incidence of dengue and chikungunya in Rio de Janeiro, Brazil: a spatiotemporal modelling study. Lancet Infect Dis. 2022; 22: 1587–1595. https://doi.org/10.1016/S1473-3099(22)00436-4 PMID: 36182679
18. World Health Organization. WHO statement on the first meeting of the International Health Regulations (2005) (IHR 2005) Emergency Committee on Zika virus and observed increase in neurological

disorders and neonatal malformations. [cited 15 Feb 2023]. Available: https://www.who.int/news/item/01-02-2016-who-statement-on-the-first-meeting-of-the-international-health-regulations-(2005)-(ihr-2005)-emergency-committee-on-zika-virus-and-observed-increase-in-neurological-disorders-and-neonatal-malformations

19. World Health Organization. Vector control operations framework for Zika virus. World Health Organization; 2016. Report No.: WHO/ZIKV/VC/16.4. Available: https://apps.who.int/iris/handle/10665/207481

20. Vazquez-Prokopec GM, Montgomery BL, Horne P, Clennon JA, Ritchie SA. Combining contact tracing with targeted indoor residual spraying significantly reduces dengue transmission. Sci Adv. 2017; 3: e1602024. https://doi.org/10.1126/sciadv.1602024 PMID: 28232955

21. Yeap HL, Axford JK, Popovici J, Endersby NM, Iturbe-Ormaetxe I, Ritchie SA, et al. Assessing quality of life-shortening *Wolbachia*-infected *Aedes aegypti* mosquitoes in the field based on capture rates and morphometric assessments. Parasit Vectors. 2014; 7: 58. https://doi.org/10.1186/1756-3305-7-58 PMID: 24495395

22. DANE Colombia. Proyecciones de población a nivel municipal, periodo 2018–2035. 2023 [cited 22 Feb 2023]. Available: https://www.dane.gov.co/index.php/estadisticas-por-tema/demografia-y-poblacion/proyecciones-de-poblacion

23. Instituto de Hidrología, Meteorología y Estudios Ambientales. PRINCIPAL—IDEAM. [cited 22 Mar 2023]. Available: http://www.ideam.gov.co/web/tiempo-y-clima/tiempo-clima

24. Costa GB, Smithyman R, O'Neill SL, Moreira LA. How to engage communities on a large scale? Lessons from World Mosquito Program in Rio de Janeiro, Brazil. Gates Open Res. 2021;4. https://doi.org/10.12688/gatesopenres.13153.2 PMID: 33103066

25. Hien NT, Anh DD, Le NH, Yen NT, Phong TV, Nam VS, et al. Environmental factors influence the local establishment of *Wolbachia* in *Aedes aegypti* mosquitoes in two small communities in central Vietnam. Gates Open Research. 2022. p. https://doi.org/10.12688/gatesopenres.13347.2 PMID: 35602266

26. World Health Organization. Test procedures for insecticide resistance monitoring in malaria vector mosquitoes. World Health Organization; 2016. Available: https://apps.who.int/iris/handle/10665/250677

27. Moyes CL, Vontas J, Martins AJ, Ng LC, Koou SY, Dusfour I, et al. Contemporary status of insecticide resistance in the major *Aedes* vectors of arboviruses infecting humans. PLoS Negl Trop Dis. 2017; 11: e0005625. https://doi.org/10.1371/journal.pntd.0005625 PMID: 28727779

28. Aponte A, Penilla RP, Rodríguez AD, Ocampo CB. Mechanisms of Pyrethroid Resistance in *Aedes (Stegomyia) aegypti* from Colombia. Acta Trop. 2019; 191: 146–154. https://doi.org/10.1016/j.actatropica.2018.12.021 PMID: 30552882

29. Granada Y, Mejía-Jaramillo AM, Zuluaga S, Triana-Chávez O. Molecular surveillance of resistance to pyrethroids insecticides in Colombian *Aedes aegypti* populations. PLoS Negl Trop Dis. 2021; 15: e0010001. https://doi.org/10.1371/journal.pntd.0010001 PMID: 34905537

30. Sombié A, Saiki E, Yaméogo F, Sakurai T, Shirozu T, Fukumoto S, et al. High frequencies of F1534C and V1016I kdr mutations and association with pyrethroid resistance in *Aedes aegypti* from Somgandé (Ouagadougou), Burkina Faso. Trop Med Health. 2019; 47: 2. https://doi.org/10.1186/s41182-018-0134-5 PMID: 30787670

31. Martins AJ, Lima JBP, Peixoto AA, Valle D. Frequency of Val1016Ile mutation in the voltage-gated sodium channel gene of *Aedes aegypti* Brazilian populations. Trop Med Int Health. 2009; 14: 1351–1355. https://doi.org/10.1111/j.1365-3156.2009.02378.x PMID: 19735371

32. Li C-X, Kaufman PE, Xue R-D, Zhao M-H, Wang G, Yan T, et al. Relationship between insecticide resistance and kdr mutations in the dengue vector *Aedes aegypti* in Southern China. Parasit Vectors. 2015; 8: 325. https://doi.org/10.1186/s13071-015-0933-z PMID: 26068925

33. Aliota MT, Peinado SA, Velez ID, Osorio JE. The *w*Mel strain of *Wolbachia* Reduces Transmission of Zika virus by *Aedes aegypti*. Sci Rep. 2016; 6: 28792. https://doi.org/10.1038/srep28792 PMID: 27364935

34. Focks DA. An Improved Separator for the Developmental Stages, Sexes, and Species of Mosquitoes (Diptera: Culicidae). J Med Entomol. 1980; 17: 567–568. https://doi.org/10.1093/jmedent/17.6.567 PMID: 6111610

35. Carvalho DO, Nimmo D, Naish N, McKemey AR, Gray P, Wilke ABB, et al. Mass Production of Genetically Modified *Aedes aegypti* for Field Releases in Brazil. J Vis Exp JoVE. 2014; 3579. https://doi.org/10.3791/3579 PMID: 24430003

36. Quyen DL, Thanh Le N, Van Anh CT, Nguyen NB, Hoang DV, Montgomery JL, et al. Epidemiological, Serological, and Virological Features of Dengue in Nha Trang City, Vietnam. Am J Trop Med Hyg. 2018; 98: 402–409. https://doi.org/10.4269/ajtmh.17-0630 PMID: 29313471

37. Moreira LA, Iturbe-Ormaetxe I, Jeffery JA, Lu G, Pyke AT, Hedges LM, et al. A *Wolbachia* Symbiont in *Aedes aegypti* Limits Infection with Dengue, Chikungunya, and *Plasmodium*. Cell. 2009; 139: 1268–1278. https://doi.org/10.1016/j.cell.2009.11.042 PMID: 20064373

38. Rancès E, Johnson TK, Popovici J, Iturbe-Ormaetxe I, Zakir T, Warr CG, et al. The Toll and Imd Pathways Are Not Required for *Wolbachia*-Mediated Dengue Virus Interference. J Virol. 2013; 87: 11945–11949. https://doi.org/10.1128/JVI.01522-13 PMID: 23986574

39. Frentiu FD, Zakir T, Walker T, Popovici J, Pyke AT, Hurk A van den, et al. Limited Dengue Virus Replication in Field-Collected *Aedes aegypti* Mosquitoes Infected with *Wolbachia*. PLoS Negl Trop Dis. 2014; 8: e2688. https://doi.org/10.1371/journal.pntd.0002688 PMID: 24587459

40. Nguyen TH, Nguyen HL, Nguyen TY, Vu SN, Tran ND, Le TN, et al. Field evaluation of the establishment potential of *w*MelPop *Wolbachia* in Australia and Vietnam for dengue control. Parasit Vectors. 2015; 8: 563. https://doi.org/10.1186/s13071-015-1174-x PMID: 26510523

41. Nazni WA, Hoffmann AA, NoorAfizah A, Cheong YL, Mancini MV, Golding N, et al. Establishment of *Wolbachia* Strain *w*AlbB in Malaysian Populations of *Aedes aegypti* for Dengue Control. Curr Biol. 2019; 29: 4241–4248.e5. https://doi.org/10.1016/j.cub.2019.11.007 PMID: 31761702

42. Gouagna LC, Damiens D, Oliva CF, Boyer S, Le Goff G, Brengues C, et al. Strategic Approach, Advances, and Challenges in the Development and Application of the SIT for Area-Wide Control of *Aedes albopictus* Mosquitoes in Reunion Island. Insects. 2020; 11: 770. https://doi.org/10.3390/insects11110770 PMID: 33171885

43. Beebe NW, Pagendam D, Trewin BJ, Boomer A, Bradford M, Ford A, et al. Releasing incompatible males drives strong suppression across populations of wild and *Wolbachia* -carrying *Aedes aegypti* in Australia. Proc Natl Acad Sci. 2021; 118: e2106828118. https://doi.org/10.1073/pnas.2106828118 PMID: 34607949

44. Crawford JE, Clarke DW, Criswell V, Desnoyer M, Cornel D, Deegan B, et al. Efficient production of male *Wolbachia*-infected *Aedes aegypti* mosquitoes enables large-scale suppression of wild populations. Nat Biotechnol. 2020; 38: 482–492. https://doi.org/10.1038/s41587-020-0471-x PMID: 32265562

45. Mains JW, Kelly PH, Dobson KL, Petrie WD, Dobson SL. Localized Control of *Aedes aegypti* (Diptera: Culicidae) in Miami, FL, via Inundative Releases of *Wolbachia*-Infected Male Mosquitoes. J Med Entomol. 2019; 56: 1296–1303. https://doi.org/10.1093/jme/tjz051 PMID: 31008514

46. Kittayapong P, Ninphanomchai S, Limohpasmanee W, Chansang C, Chansang U, Mongkalangoon P. Combined sterile insect technique and incompatible insect technique: The first proof-of-concept to suppress *Aedes aegypti* vector populations in semi-rural settings in Thailand. PLoS Negl Trop Dis. 2019; 13: e0007771. https://doi.org/10.1371/journal.pntd.0007771 PMID: 31658265

47. Zheng X, Zhang D, Li Y, Yang C, Wu Y, Liang X, et al. Incompatible and sterile insect techniques combined eliminate mosquitoes. Nature. 2019; 572: 56–61. https://doi.org/10.1038/s41586-019-1407-9 PMID: 31316207

48. Martín-Park A, Che-Mendoza A, Contreras-Perera Y, Pérez-Carrillo S, Puerta-Guardo H, Villegas-Chim J, et al. Pilot trial using mass field-releases of sterile males produced with the incompatible and sterile insect techniques as part of integrated *Aedes aegypti* control in Mexico. PLoS Negl Trop Dis. 2022; 16: e0010324. https://doi.org/10.1371/journal.pntd.0010324 PMID: 35471983

49. Harris AF, McKemey AR, Nimmo D, Curtis Z, Black I, Morgan SA, et al. Successful suppression of a field mosquito population by sustained release of engineered male mosquitoes. Nat Biotechnol. 2012; 30: 828–830. https://doi.org/10.1038/nbt.2350 PMID: 22965050

50. Carvalho DO, McKemey AR, Garziera L, Lacroix R, Donnelly CA, Alphey L, et al. Suppression of a Field Population of *Aedes aegypti* in Brazil by Sustained Release of Transgenic Male Mosquitoes. PLoS Negl Trop Dis. 2015; 9: e0003864. https://doi.org/10.1371/journal.pntd.0003864 PMID: 26135160

51. Rojo-Ospina RA, Quimbayo-Forero M, Calle-Tobón A, Bedoya-Patiño SC, Gómez M, Ramírez A, et al. Integrated vector management program in the framework of the COVID-19 pandemic in Medellin, Colombia. Biomed Rev Inst Nac Salud. 2023; 43: 131–144. https://doi.org/10.7705/biomedica.6679 PMID: 37167464

52. Tantowijoyo W, Tanamas SK, Nurhayati I, Setyawan S, Budiwati N, Fitriana I, et al. *Aedes aegypti* abundance and insecticide resistance profiles in the Applying *Wolbachia* to Eliminate Dengue trial. PLoS Negl Trop Dis. 2022; 16: e0010284. https://doi.org/10.1371/journal.pntd.0010284 PMID: 35442957

53. Hancock PA, Sinkins SP, Godfray HCJ. Population Dynamic Models of the Spread of *Wolbachia*. Am Nat. 2011; 177: 323–333. https://doi.org/10.1086/658121 PMID: 21460541

54. Marinho RA, Beserra EB, Bezerra-Gusmão MA, Porto V de S, Olinda RA, dos Santos CAC. Effects of temperature on the life cycle, expansion, and dispersion of *Aedes aegypti* (Diptera: Culicidae) in three cities in Paraiba, Brazil. J Vector Ecol. 2016; 41: 1–10. https://doi.org/10.1111/jvec.12187 PMID: 27232118

55. Carrington LB, Armijos MV, Lambrechts L, Barker CM, Scott TW. Effects of Fluctuating Daily Temperatures at Critical Thermal Extremes on *Aedes aegypti* Life-History Traits. PLOS ONE. 2013; 8: e58824. https://doi.org/10.1371/journal.pone.0058824 PMID: 23520534

56. Velez ID, Tanamas SK, Arbelaez MP, Kutcher SC, Duque SL, Uribe A, et al. Reduced dengue incidence following area-wide deployments of *w*Mel-infected mosquitoes throughout three Colombian cities. PLoS Negl Trop Dis 17(11): e0011713. https://doi.org/10.1371/journal.pntd.0011713
